# Green synthesis, structure–activity relationships, *in silico* molecular docking, and antifungal activities of novel prenylated chalcones

**DOI:** 10.3389/fchem.2024.1389848

**Published:** 2024-04-26

**Authors:** Rajni Godara, Parshant Kaushik, Kailashpati Tripathi, Rakesh Kumar, Virendra Singh Rana, Rajesh Kumar, Abhishek Mandal, V. Shanmugam, Pankaj, Najam Akhtar Shakil

**Affiliations:** ^1^ Division of Agricultural Chemicals, ICAR-Indian Agricultural Research Institute, New Delhi, India; ^2^ The Graduate School, ICAR-Indian Agricultural Research Institute, New Delhi, India; ^3^ ICAR-National Research Centre on Seed Spices, Ajmer, Rajasthan, India; ^4^ ICAR-Central Inland Fisheries Research Institute, Guwahati, Assam, India; ^5^ ICAR-Indian Institute of Horticultural Research, Bengaluru, Karnataka, India; ^6^ Division of Plant Pathology, ICAR-Indian Agricultural Research Institute, New Delhi, India; ^7^ Division of Nematology, ICAR-Indian Agricultural Research Institute, New Delhi, India

**Keywords:** antifungal activity, ED_50_, *Fusarium oxysporum*, green synthesis, molecular docking, prenylated chalcones, *Sclerotium rolfsii*, structure–activity relationship

## Abstract

A series of 16 novel prenylated chalcones (**5A-5P**) was synthesized by microwave-assisted green synthesis using 5-prenyloxy-2-hydroxyacetophenone and different benzaldehydes. Comparisons were also performed between the microwave and conventional methods in terms of the reaction times and yields of all compounds, where the reaction times in the microwave and conventional methods were 1–4 min and 12–48 h, respectively. The synthesized compounds were characterized using different spectroscopic techniques, including IR, ^1^H-NMR, ^13^C-NMR, and LC-HRMS. The antifungal activities of all compounds were evaluated against *Sclerotium rolfsii* and *Fusarium oxysporum* under *in vitro* conditions and were additionally supported by structure–activity relationship (SAR) and molecular docking studies. Out of the 16 compounds screened, 2’-hydroxy-4-benzyloxy-5’-*O*-prenylchalcone (**5P**) showed the highest activity against both *S. rolfsii* and *F. oxysporum*, with ED_50_ of 25.02 and 31.87 mg/L, respectively. The molecular docking studies of the prenylated chalcones within the active sites of the EF1*α* and RPB2 gene sequences and FoCut5a sequence as the respective receptors for *S. rolfsii* and *F. oxysporum* revealed the importance of the compounds, where the binding energies of the docked molecules ranged from −38.3538 to −26.6837 kcal/mol for *S. rolfsii* and −43.400 to −23.839 kcal/mol for *F. oxysporum*. Additional docking parameters showed that these compounds formed stable complexes with the protein molecules.

## 1 Introduction

Chalcones are vital secondary metabolites sourced from various edible plants, such as fruits, vegetables, tea, and spices, as well as other natural food items. They also serve as essential intermediates in the biosynthesis of flavonoids (Iwashina, 2000; Piñero et al., 2006). The presence of a double bond between the *α* and *β* positions gives them a particularly unique molecular structure that holds significant importance in organic chemistry, owing to their versatility and reactivity. In their review, Keiko and Vchislo (2016a) summarized the recently developed methods for the synthesis of five-membered N,N-, N,O-, and N,S-heterocycles involving *α*,*β-*unsaturated carbonyl compounds. One of the notable aspects of chalcones and other *α*,*β-*unsaturated compounds is their capability to undergo various ring-closure reactions, leading to the formation of diverse heterocyclic scaffolds that serve as crucial intermediates in the synthesis of various agrochemicals, including fungicides, nematicides, herbicides, insecticides, and other pesticides (Keiko and Vchislo, 2016b). Zhang et al. (2023) reported the utilization of *α*,*β-*unsaturated carbonyl compounds in electrophilic spirocyclization to form 4-halomethyl-2-azaspiro [4.5]decanes. Tandel et al. (2022) synthesized highly fluorescent benzothiophene-based chalcone scaffolds by reacting substituted aromatic ketones and benzo [*b*]thiophene carbaldehyde in the presence of catalysts.

Years of scientific research have revealed that chalcones exhibit diverse biological activities (Rajendran et al., 2022; Hartman et al., 2024; Yousefian et al., 2024), with antioxidant (Jung et al., 2017), antimicrobial (Zhou et al., 2021), anti-inflammatory, antitumor (Neves et al., 2012), anti-infective (Nowakowska, 2007), anticancer (Binate and Ganbarov, 2023), antiviral (Hu et al., 2023), cytotoxic (Vogel and Heilmann, 2008), antiallergic (Escribano-Ferrer et al., 2019), antituberculosis (Verma et al., 2018), and antibacterial (Nielsen et al., 2005; Wang et al., 2023; Zhang et al., 2024) properties. Chalcones are small, achiral molecules that have molecular weights in the range of 300–600 g/mol and exhibit relatively high lipophilicities (Nowakowska, 2007).

**FIGURE 1:**
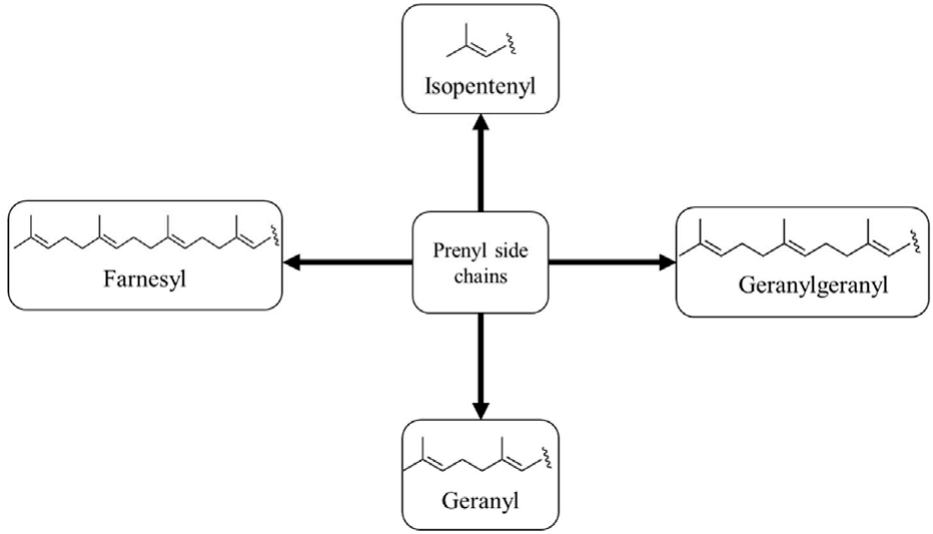
Different prenyl side chains.

**FIGURE 2:**
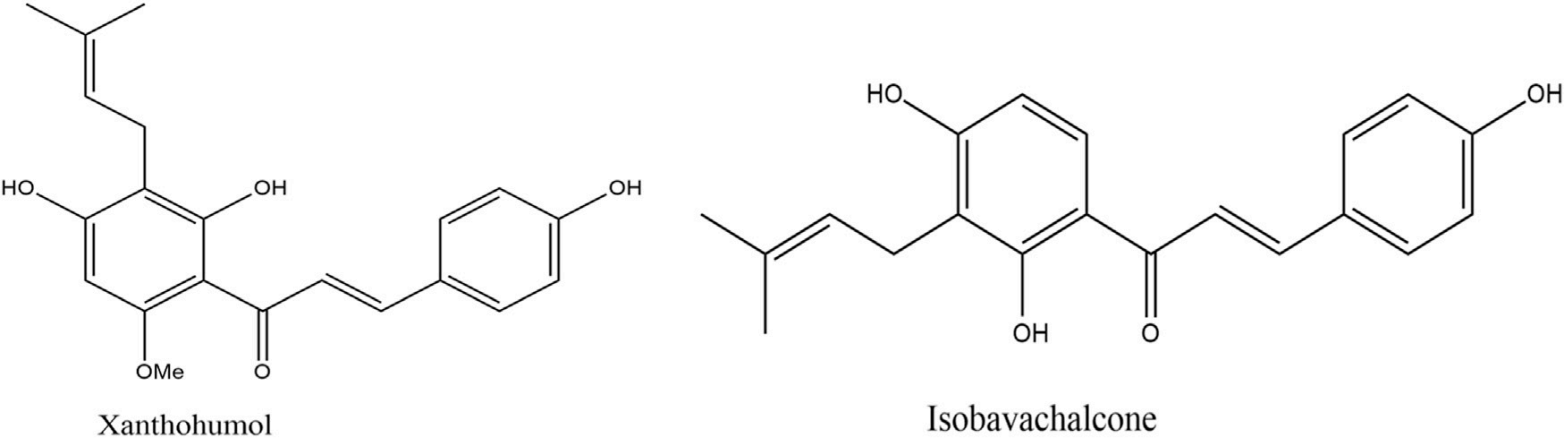
Some naturally occurring prenylated chalcones.

Prenylation is a method in which there is a chemical or enzymatic addition of a hydrophobic side chain (C5 isoprene units) to an accepting molecule (another terpenoid molecule, an aromatic compound, a protein, etc.) (Figure 1) (Passalacqua et al., 2015). Chalcones with a prenyl side chain on the skeleton are classified as prenylated chalcones. Frequently, the addition of an isoprenoid chain renders the molecule more effective than the parent compound. However, the incorporation of a prenyl fragment into the chalcone scaffold is limited to the aromatic rings, which has led to various designs and synthesis methods of these compounds (Jin et al., 2014). Their simple structure and easy preparation make chalcones attractive scaffolds for the synthesis of a large number of derivatives. Chalcones with modified or unmodified prenyl moieties on rings A and B have been discovered in plants of the Fabaceae, Moraceae, Zingiberaceae, and Cannabaceae families (Zhou et al., 2021). Some of the notable examples include xanthohumol and isobavachalcone (Figure 2), which have been studied extensively for their diverse biological activities. However, prenylated chalcones have limited availability in nature because of their low content in natural sources, availability in complex mixtures in plant extracts, and lack of economical production systems (Yazaki et al., 2009).

Claisen–Schmidt condensation is a widely used method for the synthesis of chalcones, including prenylated chalcones. There are many reports on synthesizing prenylated chalcones via conventional methods like refluxing (Vogel and Heilmann, 2008; Dong et al., 2009; Sugamoto et al., 2011; Passalacqua et al., 2015; Espinoza-Hicks et al., 2019; Rani et al., 2019) and stirring (Neves et al., 2012; Osorio et al., 2016). However, these methods are not without limitations, with the primary disadvantages being their slow reaction rates, lower yields, and formation of byproducts (Gaonkar and Vignesh, 2017). Microwave heating has been established as an incontestable method of activation in analytical and organic laboratory practices, especially for its efficacy and environment-friendly nature. The rate enhancements in these organic reactions can be attributed to the rapid superheating of the solvent, which facilitates faster reaction kinetics (Gedye et al., 1986). Reactions like the Diels–Alder, Claisen condensation, and Ene reactions (Giguere et al., 1986) are well-suited for microwave-assisted organic synthesis (MAOS) as they can be performed in significantly less time than conventional methods while still providing good yields of the desired products. Scientists are gradually focusing more on ecofriendly methods of chemical syntheses, with particular attention to designing efficient catalysts and advancing green chemistry principles. In recent times, a number of chalcone derivatives have been synthesized utilizing ionic liquids (ILs) as both the solvents and catalysts for the ring-closure reactions of chalcones (Dhadda et al., 2022). Moreover, nanocatalysts have been utilized as green alternatives to conventional catalysts in the synthesis of numerous chalcone derivatives (Jain et al., 2021). Green methods have also been utilized in the medical field, such as the use of ultrasound to observe mycosis fungoides (Chen et al., 2022). Hence, there is a gradual shift toward green chemical synthesis in producing various chalcone derivatives for diverse applications.

Fungal plant diseases have substantial and far-reaching influences on food security, which is a matter of global concern. These diseases induce changes across the developmental stages, resulting in substantial losses and issues related to aspects like quality, nutritional value, and shelf life (Agrios, 2004). Root rot disease caused by the soilborne fungus *Sclerotium rolfsii* (Yanti et al., 2021) and Fusarium wilt caused by *Fusarium oxysporum* are significant and devastating problems for farmers across the globe (McGovern, 2015). Chemical interventions have always played crucial roles in the effective management and prevention of fungal diseases in plants. However, such methods have also been associated with resistance development and potential adverse effects on human health and the environment (Dhankhar and Kumar, 2023). Consequently, there is a need to develop compounds with low dosage, high potency, environmentally friendly characteristics, and reduced persistence in the ecosystem. Recent innovations in biological research, notably metabolomics and genomics, hold great promise for advancing agricultural research; they offer the means to search for new potential agrochemical fungicides and also help in finding the key compounds responsible for antifungal activities (Wang et al., 2020; Alawiye and Babalola, 2021; Jiang et al., 2023). *S. rolfsii* exhibits various populations that can be categorized into mycelial compatibility groups (MCGs). Two protein-coding genes, namely, translation elongation factor 1α (EF1α) and RNA polymerase II subunit two (RPB2), can be successfully used to understand the epidemiology of Sclerotium root rot diseases (Remesal et al., 2013).

Chalcones offer significant potential as bioactive agents because of their simple structures and versatile functionalization capacities. Although chalcone synthesis using microwave irradiation has been documented extensively, to the best of the authors’ knowledge, there are no prior reported attempts for synthesizing prenylated chalcones using this technique. In this ongoing pursuit of synthesizing bioactive molecules, the synthesis of novel prenylated chalcones by employing an environment friendly but high-yield and rapid method, such as microwave irradiation, is presented along with characterizations by different spectroscopic techniques, such as IR, ^1^H-NMR, ^13^C-NMR, and LC-HRMS. The synthesized compounds are also evaluated for their potential as antifungal agents against *S. rolfsii* and *F. oxysporum.* Additionally, structure–activity relationship (SAR) and molecular docking studies are conducted to gain further insights into the antifungal potential of these compounds.

## 2 Experimental

### 2.1 Chemicals and instruments

Prenyl bromide as well as various acetophenones and benzaldehydes were purchased from Sigma-Aldrich and used as received unless specified otherwise. All solvents and chemicals used were of analytical grade. The reactions were monitored using thin-layer chromatography (TLC) on Merck silica gel 60F_254_ with 200-mm-thick aluminum sheets and visualized under ultraviolet (UV) light. The purity of each synthesized prenylated chalcone was determined by UFLC-PDA (SHIMADZU) using a C-18 Shim-pack column (5 µm, 4.6 × 250 mm) and methanol: water in the ratio of 98:2 in an isocratic solvent system. The ^1^H-NMR and ^13^C-NMR spectra were recorded using a 400-MHz Spectrospin spectrometer (JEOL) instrument, and tetramethylsilane (TMS) was used as an internal standard. The chemical shift values were on the *δ* scale, coupling constant J was in terms of hertz, and data were processed using Delta software. Accurate masses of the compounds were determined by LC-HRMS (AB SCIEX Triple TOFTM 5600^+^) equipped with TurboIonSpray (TIS) and SCIEX ExionLC coupled with a PDA detector and C-18 column (2.7 µm, 4.6 × 100 mm). The column was eluted with methanol and water in the ratio of 98:2 with 0.1% formic acid at a flow rate of 0.5 mL/min. The column oven temperature was set at 40°C; infrared (IR) spectra were recorded using a Fourier-transform infrared (FT-IR) spectrophotometer (Bruker Alpha), and the melting points were determined using a melting point apparatus. The ED_50_ values were calculated using SPSS statistical software (v16.0).

### 2.2 Synthesis

#### 2.2.1 Conventional method (CM)

**SCHEME 1:**
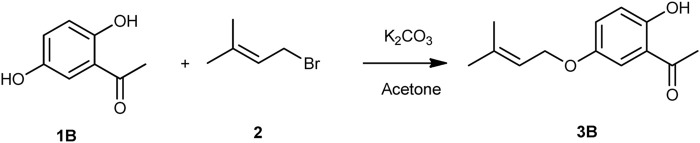
Synthesis scheme of 5-prenyloxy-2-hydroxyacetophenone **(3B)**.

**SCHEME 2:**
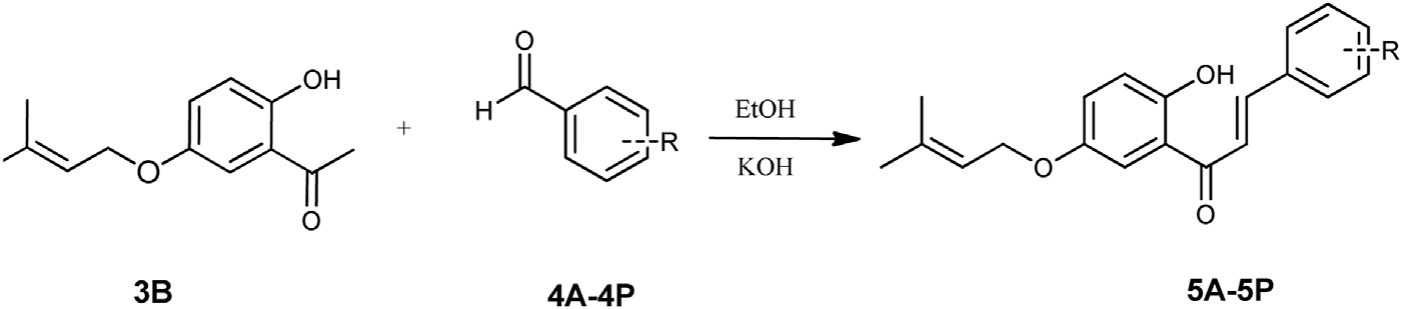
Synthesis scheme of the prenylated chalcones **(5A-5P)**.

The prenylated chalcones were synthesized in two steps by the CM reported in the literature (Reddy et al., 2010; Rani et al., 2019). For the synthesis of prenylated acetophenone (**3B**), freshly ignited K_2_CO_3_ (2 g) and prenyl bromide (**2**) (96 mg, 0.657 mmol) were added to a solution of dihydroxyacetophenone (**1B**) (100 mg, 0.657 mmol) in acetone (10 mL) in the molar ratio of 1:1. The reaction mixture was refluxed for 5–20 h, and upon reaction completion, the reaction mixture was filtered and solvent was evaporated. The residue was purified by silica gel column chromatography and eluted with hexane: ethyl acetate (4:1) to obtain the required compound (Scheme 1). For preparation of the prenylated chalcones, prenylated acetophenone (**3B**) (100 mg, 0.454 mmol) was taken in 40% KOH solution (5 mL) in a round-bottom flask, followed by dropwise addition of ethanolic solutions (10 mL) of different benzaldehydes (**4A-4P**) in different ratios with continuous stirring. The reaction mixtures were then refluxed at 60°C for 12–48 h each, and the reactions were monitored using TLC in the hexane: ethyl acetate (9:1) solvent system. After reaction completion, each reaction mixture was neutralized with 2M HCl to obtain a precipitate, which was filtered and washed with cold water to obtain the final product (**5A-5P**). The final products were purified by silica gel column chromatography and crystallized in methanol (Scheme 2).

#### 2.2.2 Microwave method (MM)

In this method, 40% KOH solution (5 mL) of prenylated acetophenone (100 mg, 0.454 mmol) was taken in a 20 mL vial, and ethanolic solutions (10 mL) of various benzaldehydes in different ratios were added and placed in the laboratory microwave oven. In the microwave, 10–15 heating cycles of 5 s each were applied with cooling in between. The total reaction time ranged from 1.5 to 4 min per compound. The reaction progress was monitored by TLC using the hexane: ethyl acetate (9:1) solvent system. After reaction completion, each reaction mixture was worked up in a manner similar to that in the CM (Section 2.2.1). The physical data on all the synthesized prenylated chalcones are presented in Table 1.

**TABLE 1:** Physical data on all the synthesized prenylated chalcones.

S. no.	Compound	Molecular formula	Molecular weight	Melting point	R_f_ value
				°C	
1	**5A**	C_20_H_19_NO_5_	353.57	102–104	0.58
2	**5B**	C_20_H_18_Cl_2_O_3_	377.26	88–90	0.53
3	**5C**	C_20_H_18_Cl_2_O_3_	377.26	98–100	0.49
4	**5D**	C_20_H_19_ClO_3_	342.82	96–98	0.60
5	**5E**	C_20_H_19_BrO_3_	387.27	98–100	0.57
6	**5F**	C_20_H_19_BrO_3_	387.27	96–98	0.60
7	**5G**	C_20_H_19_BrO_3_	387.27	99–101	0.66
8	**5H**	C_20_H_19_NO_5_	353.57	111–113	0.52
9	**5I**	C_23_H_26_O_6_	398.45	97–99	0.50
10	**5J**	C_20_H_19_NO_5_	353.57	105–107	0.48
11	**5K**	C_20_H_20_O_4_	324.37	97–99	0.55
12	**5L**	C_20_H_20_O_4_	324.37	99–101	0.57
13	**5M**	C_22_H_24_O_4_	352.42	97–99	0.52
14	**5N**	C_21_H_22_O_4_	338.40	98–100	0.58
15	**5O**	C_22_H_25_NO_3_	351.44	94–96	0.55
16	**5P**	C_27_H_26_O_4_	415.19	97–99	0.59

Bold values represent the codes for compounds.

### 2.3 Spectral analyses of the synthesized prenylated chalcones **(5A-5P)**

#### 2.3.1 2’-Hydroxy-3-nitro-5’-*O*-prenylchalcone **(5A)**

Orange solid. IR (KBr, cm^-1^): 1,677 (C=O), 1,562 (CH=CH); ^1^H-NMR (400 MHz, CDCl_3_): *δ* 1.73 (3H, s, prenyl-CH_3_), 1.80 (3H, s, prenyl-CH_3_), 4.54 (2H, d, J = 6.8 Hz, H-1”), 5.49 (1H, t, J = 6.8 Hz, H-2”), 6.97 (1H, d, J = 9.2 Hz, H-3’), 7.08 (1H, dd, J = 9.2 Hz and 3.2 Hz, H-4’), 7.33 (1H, d, J = 3.2 Hz, H-6’), 7.57 (1H, t, J = 8 Hz and 8 Hz, H-5), 7.67 (1H, d, J = 16 Hz, H-*α*), 7.75 (1H, d, J = 16 Hz, H-*β*), 7.86 ((1H, d, J = 8 Hz, H-6), 8.22 (1H, m, H-4), 8.44 (1H, m, H-2), and 12.18 (1H, s, chelated OH); ^13^C-NMR (100 MHz, CDCl_3_): *δ* 18.21 (prenyl-CH_3_), 25.75 (prenyl-CH_3_), 65.38 (C-1”), 115.09 (C-3’ and C-4’), 119.11 (C-2’’), 119.44 (C-1’), 121.52 (C-6), 122.21 (C-4), 124.14 (C-2), 129.85 (C-*α*, C-5, and C-6’), 134.08 (C-1), 137.29 (C-3), 138.68 (C-3’’), 148.66 (C-*β*), 152.65 (C-5’), 152.98 (C-2’), and 190.82 (C=O). HR-MS for C_20_H_19_NO_5_ [M + H]^+^ *m/z*: Calcd 354.1336; observed 354.1733.

#### 2.3.2 2’-Hydroxy-2,6-dichloro-5’-*O*-prenylchalcone **(5B)**

Bright orange solid. IR (KBr, cm^-1^): 1,647 (C=O), 1,549 (CH=CH); ^1^H-NMR (400 MHz, CDCl_3_): *δ* 1.73 (3H, s, prenyl-CH_3_), 1.79 (3H, s, prenyl-CH_3_), 4.49 (2H, d, J = 6.8 Hz, H-1”), 5.47 (1H, t, J = 6.8 Hz, H-2”), 6.97 (1H, d, J = 8.8 Hz, H-3’), 7.17 (1H, dd, J = 8.8 Hz and 2.8 Hz, H-4’), 7.23(1H, m, H-4), 7.31 (1H, d, J = 2.8 Hz, H-6’), 7.40 (2H, d, J = 8 Hz, H-5 and H-3), 7.75 (1H, d, J = 16 Hz, H-*α*), 7.95 (1H, d, J = 16 Hz, H-*β*), and 12.20 (1H, s, chelated OH); ^13^C-NMR (100 MHz, CDCl_3_): *δ* 18.19 (prenyl-CH_3_), 25.78 (prenyl-CH_3_), 65.65 (C-1”), 101.66 (C-3’), 108.12 (C-4’), 113.95 (C-1’), 117.77 (C-*α*), 118.54 (C-2”), 128.93 (C-1), 129.89 (C-3 and C-5), 131.49 (C-6’), 132.41 (C-4), 135.22 (C-2 and C-6), 139.15 (C-3’’), 144.12 (C-*β*), 150.90, (C-5’), 157.95 (C-2’), and 193.29 (C=O). HR-MS for C_20_H_18_Cl_2_O_3_ [M + H]^+^ *m/z*: Calcd 377.0705; observed 377.1002.

#### 2.3.3 2’-Hydroxy-2,4-dichloro-5’-*O*-prenylchalcone **(5C)**

Orange solid. IR (KBr, cm^-1^): 1,677 (C=O), 1,564 (CH=CH); ^1^H-NMR (400 MHz, CDCl_3_): *δ* 1.75 (3H, s, prenyl-CH_3_), 1.81 (3H, s, prenyl-CH_3_), 4.51 (2H, d, J = 6.8 Hz, H-1”), 5.49 (1H, t, J = 6.8 Hz, H-2”), 6.98 (1H, d, J = 8.8 Hz, H-3’), 7.18 (1H, dd, J = 8.8 Hz and 3.2 Hz, H-4’), 7.32 (1H, dd, J = 8.8 Hz and 2.8 Hz, H-5), 7.35 (1H, d, J = 2.8 Hz, H-3), 7.49 (1H, d, J = 3.2 Hz, H-6’), 7.54 (1H, d, J = 15.6 Hz, H-*α*), 7.68 (1H, d, J = 8.8 Hz, H-6), 8.20 (1H, d, J = 16 Hz, H-*β*), and 12.21 (1H, s, chelated OH); ^13^C-NMR (100 MHz, CDCl_3_): *δ* 18.21 (prenyl-CH_3_), 25.80 (prenyl-CH_3_), 65.72(C-1”), 114.27 (C-3’), 119.26 (C-4’), 119.38 (C-1’), 123.06 (C-*α*) 124.78 (C-2”), 127.57 (C-1), 128.50 (C-6), 130.18 (C-5), 131.45 (C-6’), 136.22 (C-2), 136.82 (C-3), 138.68 (C-4), 139.77 (C-3’’), 144.12 (C-*β*), 150.89, (C-5’), 157.89 (C-2’), and 192.82 (C=O). HR-MS for C_20_H_18_Cl_2_O_3_ [M + H]^+^ *m/z*: Calcd 377.0705; observed 377.0768.

#### 2.3.4 2’-Hydroxy-3-chloro-5’-*O*-prenylchalcone **(5D)**

Red solid. IR (KBr, cm^-1^): 1,677 (C=O), 1,546 (CH = CH); ^1^H-NMR (400 MHz, CDCl_3_): *δ* 1.77 (3H, s, prenyl-CH_3_), 1.82 (3H, s, prenyl-CH_3_), 4.53 (2H, d, J = 6.8 Hz, H-1”), 5.50 (1H, t, J = 6.8 Hz, H-2”), 6.97 (1H, d, J = 9.2 Hz, H-3’), 7.17 (1H, dd, J = 9.2 Hz and 3.2 Hz, H-4’), 7.40 (3H, m, H-6, H-5, and H-4), 7.51 (1H, m, H-2), 7.57 (1H, d, J = 15.6 Hz, H-*α*), 7.64 (1H, m, H-6’), 7.82 (1H, d, J = 16 Hz, H-*β*), and 12.26 (1H, s, chelated OH); ^13^C-NMR (100 MHz, CDCl_3_): *δ* 18.21 (prenyl-CH_3_), 25.79 (prenyl-CH_3_), 65.22 (C-1”), 117.61 (C-3’), 119.45 (C-4’), 122.38 (C-1’), 125.88 (C-*α*), 126.82 (C-2”), 127.39 (C-1), 128.28 (C-6), 128.85 (C-6’), 132.95 (C-5), 133.23 (C-2), 134.64 (C-4), 136.37 (C-3’’), 138.42 (C-*β*), 141.78 (C-3), 150.81, (C-5’), 156.19 (C-2’), and 191.25 (C=O). HR-MS for C_20_H_19_ClO_3_ [M + H]^+^ *m/z*: Calcd 343.1095; observed 343.0899.

#### 2.3.5 2’-Hydroxy-4-bromo-5’-*O*-prenylchalcone **(5E)**

Bright red solid. IR (KBr, cm^-1^): 1,677 (C=O), 1,531 (CH = CH); ^1^H-NMR (400 MHz, CDCl_3_): *δ* 1.76 (3H, s, prenyl-CH_3_), 1.81 (3H, s, prenyl-CH_3_), 4.51 (2H, d, J = 6.8 Hz, H-1”), 5.49 (1H, t, J = 6.8 Hz, H-2”), 6.96 (1H, d, J = 9.2 Hz, H-3’), 7.16 (1H, dd, J = 9.2 Hz and 2.8 Hz, H-4’), 7.37 (1H, d, J = 2.8 Hz, H-6’), 7.51 (2H, m, H-6 and H-2), 7.55 (2H, m, H-5 and H-3), 7.56 (1H, d, J = 15.2 Hz, H-*α*), 7.83 (1H, d, J = 16 Hz, H-*β*), and 12.26 (1H, s, chelated OH); ^13^C-NMR (100 MHz, CDCl_3_): *δ* 18.24 (prenyl-CH_3_), 25.85 (prenyl-CH_3_), 65.75 (C-1”), 114.37 (C-1’), 119.22 (C-3’), 119.40 (C-2 and C-6), 119.57 (C-2’’), 120.68 (C-4’), 124.56 (C-*α*), 125.25 (C-6’), 129.92 (C-1), 132.28 (C-3 and C-5), 133.45 (C-4), 138.76 (C-3’’), 144.01 (C-*β*), 150.89 (C-5’), 157.85 (C-2’), and 193.08 (C=O). HR-MS for C_20_H_19_BrO_3_ [M + H]^+^ *m/z*: Calcd 387.0590; observed 387.0599.

#### 2.3.6 2’-Hydroxy-3-bromo-5’-*O*-prenylchalcone **(5F)**

Yellow solid. IR (KBr, cm^-1^): 1,676 (C=O), 1,549 (CH = CH); ^1^H-NMR (400 MHz, CDCl_3_): *δ* 1.78 (3H, s, prenyl-CH_3_), 1.82 (3H, s, prenyl-CH_3_), 4.53 (2H, d, J = 6.8 Hz, H-1”), 5.50 (1H, t, J = 6.8 Hz, H-2”), 6.97 (1H, d, J = 9.2 Hz, H-3’), 7.17 (1H, dd, J = 9.2 Hz and 2.8 Hz, H-4’), 7.32 (1H, t, J = 8 Hz and 7.6 Hz, H-5), 7.37 (1H, d, J = 2.8 Hz, H-6’), 7.55 (2H, m, H-6 and H-4), 7.56 (1H, d, J = 16 Hz, H-*α*), 7.80 (1H, m, H-2), 7.81 (1H, d, J = 16 Hz, H-*β*), and 12.26 (1H, s, chelated OH); ^13^C-NMR (100 MHz, CDCl_3_): *δ* 18.26 (prenyl-CH_3_), 25.85 (prenyl-CH_3_), 65.80 (C-1”), 114.31 (C-1’), 119.47 (C-3’ and C-4’), 121.45 (C-2’’), 123.15 (C-6), 124.47 (C-4), 124.76 (C-2), 130.70 (C-*α*, C-5, and C-6’), 133.58 (C-1), 136.64 (C-3), 138.69 (C-3’’), 143.58 (C-*β*), 150.93 (C-5’), 157.89 (C-2’), and 192.97 (C=O). HR-MS for C_20_H_19_BrO_3_ [M + H]^+^ *m/z*: Calcd 387.0590; observed 387.0891.

#### 2.3.7 2’-Hydroxy-2-bromo-5’-*O*-prenylchalcone **(5G)**

Yellow solid. IR (KBr, cm^-1^): 1,677 (C=O), 1,546 (CH = CH); ^1^H-NMR (400 MHz, CDCl_3_): *δ* 1.73 (3H, s, prenyl-CH_3_), 1.79 (3H, s, prenyl-CH_3_), 4.52 (2H, d, J = 6.8 Hz, H-1”), 5.47 (1H, t, J = 6.8 Hz, H-2”), 6.94 (1H, d, J = 9.2 Hz, H-3’), 7.05 (1H, dd, J = 9.2 Hz and 2.8 Hz, H-4’), 7.21 (1H, ddd, J = 8 Hz, 7.6 Hz, and 1.6 Hz, H-5), 7.31 (2H, m, H-6’ and H-4), 7.52 (1H, d, J = 16 Hz, H-*α*), 7.61 (1H, dd, J = 8 Hz and 1.2 Hz, H-6), 7.68 (1H, dd, J = 8 Hz and 1.6 Hz, H-3), 8.00 (1H, d, J = 16 Hz, H-*β*), and 12.30 (1H, s, chelated OH); ^13^C-NMR (100 MHz, CDCl_3_): *δ* 18.18 (prenyl-CH_3_), 25.70 (prenyl-CH_3_), 65.90 (C-1”), 115.02 (C-1’), 115.19 (C-3’), 119.36 (C-2’’), 119.41 (C-4’), 120.99 (C-3 and C-4), 125.82 (C-*α*), 127.56 (C-1), 129.47 (C-2), 130.85 (C-5 and C-6’), 135.33 (C-6), 138.44 (C-3’’), 140.53 (C-*β*), 152.35 (C-5’), 152.88 (C-2’), and 191.51 (C=O). HR-MS for C_20_H_19_BrO_3_ [M + H]^+^ *m/z*: Calcd 387.0590; observed 387.0648.

#### 2.3.8 2’-Hydroxy-4-nitro-5’-*O*-prenylchalcone **(5H)**

Red solid. IR (KBr, cm^-1^): 1,677 (C=O), 1,565 (CH = CH); ^1^H-NMR (400 MHz, CDCl_3_): *δ* 1.76 (3H, s, prenyl-CH_3_), 1.82 (3H, s, prenyl-CH_3_), 4.51 (2H, d, J = 6.8 Hz, H-1”), 5.50 (1H, t, J = 6.8 Hz, H-2”), 6.96 (1H, d, J = 9.2 Hz, H-3’), 7.16 (1H, dd, J = 9.2 Hz and 2.8 Hz, H-4’), 7.38 (1H, d, J = 2.8 Hz, H-6’), 7.52 (2H, m, H-6 and H-2), 7.55 (2H, m, H-5 and H-3), 7.57 (1H, d, J = 15.2 Hz, H-*α*), 7.83 (1H, d, J = 16 Hz, H-*β*), and 12.26 (1H, s, chelated OH); ^13^C-NMR (100 MHz, CDCl_3_): *δ* 18.24 (prenyl-CH_3_), 25.85 (prenyl-CH_3_), 65.75 (C-1”), 114.37 (C-1’), 119.22 (C-3’), 119.41 (C-2 and C-6), 119.57 (C-2’’), 120.68 (C-4’), 124.56 (C-*α*), 125.25 (C-6’), 129.92 (C-1), 132.28 (C-3 and C-5), 133.45 (C-4), 138.76 (C-3’’), 144.01 (C-*β*), 150.89 (C-5’), 157.85 (C-2’), and 193.08 (C=O). HR-MS for C_20_H_19_NO_5_ [M + H]^+^ *m/z*: Calcd 354.1336; observed 354.1733.

#### 2.3.9 2’-Hydroxy-3,4,5-trimethoxy-5’-*O*-prenylchalcone **(5I)**

Yellow solid. IR (KBr, cm^-1^): 1,677 (C=O), 1,562 (CH = CH); ^1^H-NMR (400 MHz, CDCl_3_): *δ* 1.76 (3H, s, prenyl-CH_3_), 1.81 (3H, s, prenyl-CH_3_), 3.87 (9H, s, 3 × OMe), 4.57 (2H, d, J = 6.8 Hz, H-1”), 5.49 (1H, t, J = 6.8 Hz, H-2”), 6.50 (2H, m, H-4’ and H-3’), 6.872 (2H, m, H-6 and H-2), 7.45 (1H, d, J = 15.2 Hz, H-*α*), 7.80 (1H, d, J = 15.2 Hz, H-*β*), 7.83 (1H, d, J = 8.4 Hz, H-6’), and 13.39 (1H, s, chelated OH); ^13^C-NMR (100 MHz, CDCl_3_): *δ* 18.23 (prenyl-CH_3_), 25.81 (prenyl-CH_3_), 56.22 (2 × OMe), 61.00 (OMe), 65.18 (C-1”), 101.64 (C-3’), 105.73 (C-2 and C-6), 108.31 (C-4’), 113.91 (C-1’), 118.58 (C-2’’), 119.47 (C-*α*), 130.27 (C-1), 131.11 (C-6’), 139.25 (C-3’’), 140.51 (C-4), 144.45 (C-*β*), 153.47 (C-3 and C-5), 165.53 (C-5’), 166.63 (C-2’), and 191.54 (C=O). HR-MS for C_23_H_26_O_6_ [M + H]^+^ *m/z*: Calcd 399.1802; observed 399.1882.

#### 2.3.10 2’-Hydroxy-2-nitro-5’-*O*-prenylchalcone **(5J)**

Yellow solid. IR (KBr, cm^-1^): 1,677 (C=O), 1,562 (CH = CH); ^1^H-NMR (400 MHz, CDCl_3_): *δ* 1.73 (3H, s, prenyl-CH_3_), 1.82 (3H, s, prenyl-CH_3_), 4.55 (2H, d, J = 6.8 Hz, H-1”), 5.45 (1H, t, J = 6.8 Hz, H-2”), 6.92 (1H, d, J = 9.2 Hz, H-3’), 7.04 (1H, dd, J = 9.2 Hz and 3.2 Hz, H-4’), 7.25 (1H, ddd, J = 8 Hz, 7.2 Hz, and 1.6 Hz, H-5), 7.31 (2H, m, H-4 and H-6’), 7.49 (1H, d, J = 15.6 Hz, H-*α*), 7.63 (1H, dd, J = 8 Hz and 2 Hz, H-6), 7.72 (1H, dd, J = 8 Hz and 1.6 Hz, H-3), 8.20 (1H, d, J = 15.6 Hz, H-*β*), and 13.34 (1H, s, chelated OH); ^13^C-NMR (100 MHz, CDCl_3_): *δ* 18.18 (prenyl-CH_3_), 25.70 (prenyl-CH_3_), 65.90 (C-1”), 115.02 (C-1’), 115.20 (C-3’), 119.36 (C-2”), 119.42 (C-4’), 120.98 (C-3 and C-4), 125.82 (C-*α*), 127.56 (C-1), 129.47 (C-2), 130.85 (C-5 and C-6’), 135.34 (C-6), 138.44 (C-3’’), 140.54 (C-*β*), 152.35 (C-5’), 152.88 (C-2’), and 191.51 (C=O). HR-MS for C_20_H_19_NO_5_ [M + H]^+^ *m/z*: Calcd 354.1336; observed 354.1733.

#### 2.3.11 2’-Hydroxy-4-hydroxy-5’-*O*-prenylchalcone **(5K)**

Yellow solid. IR (KBr, cm^-1^): 1,677 (C=O), 1,562 (CH = CH); ^1^H-NMR (400 MHz, CDCl_3_): *δ* 1.75 (3H, s, prenyl-CH_3_), 1.82 (3H, s, prenyl-CH_3_), 4.52 (2H, d, J = 6.8 Hz, H-1”), 5.50 (1H, t, J = 6.8 Hz, H-2”), 6.96 (1H, d, J = 9.2 Hz, H-3’), 7.16 (1H, dd, J = 9.2 Hz and 2.8 Hz, H-4’), 7.38 (1H, d, J = 2.8 Hz, H-6’), 7.52 (2H, m, H-6 and H-2), 7.56 (2H, m, H-5 and H-3), 7.57 (1H, d, J = 15.2 Hz, H-*α*), 7.84 (1H, d, J = 16 Hz, H-*β*), and 12.26 (1H, s, chelated OH); ^13^C-NMR (100 MHz, CDCl_3_): *δ* 18.24 (prenyl-CH_3_), 25.85 (prenyl-CH_3_), 65.75 (C-1”), 114.37 (C-1’), 119.22 (C-3’), 119.40 (C-2 and C-6), 119.57 (C-2’’), 120.68 (C-4’), 124.56 (C-*α*), 125.25 (C-6’), 129.92 (C-1), 132.28 (C-3 and C-5), 133.45 (C-4), 138.76 (C-3’’), 144.01 (C-*β*), 150.89 (C-5’), 157.85 (C-2’), and 193.08 (C=O). HR-MS for C_20_H_20_O_4_ [M + H]^+^ *m/z*: Calcd 325.1434; observed 325.1256.

#### 2.3.12 2’-Hydroxy-3-hydroxy-5’-*O*-prenylchalcone **(5L)**

Yellow solid. IR (KBr, cm^-1^): 1,677 (C=O), 1,567 (CH = CH); ^1^H-NMR (400 MHz, CDCl_3_): *δ* 1.73 (3H, s, prenyl-CH_3_), 1.78 (3H, s, prenyl-CH_3_), 4.52 (2H, d, J = 6.8 Hz, H-1”), 5.47 (1H, t, J = 6.8 Hz, H-2”), 6.87 (1H, m, H-6), 6.94 (1H, d, J = 9.2 Hz, H-3’), 7.03 (1H, dd, J = 9.2 Hz and 3.2 Hz, H-4’), 7.11 (1H, d, J = 16 Hz, H-*α*), 7.14 (1H, d, J = 3.2 Hz, H-6’), 7.24 (2H, m, H-4 and H-2), 7.55 (1H, d, J = 16 Hz, H-*β*) 7.61 (1H, d, J = 3.2 Hz, H-5), and 12.62 (1H, s, chelated OH); ^13^C-NMR (100 MHz, CDCl_3_): *δ* 18.14 (prenyl-CH_3_), 25.70 (prenyl-CH_3_), 65.47 (C-1”), 114.32 (C-1’), 115.34 (C-3’ and C-4’), 117.57 (C-2’’), 119.37 (C-6), 120.41 (C-4), 120.95 (C-2), 127.16 (C-1), 129.61 (C-*α*, C-5, and C-6’), 136.57 (C-3), 138.48 (C-3’’), 143.19 (C-*β*), 152.60 (C-5’), 156.49 (C-2’), and 192.71 (C=O). HR-MS for C_20_H_20_O_4_ [M + H]^+^ *m/z*: Calcd 325.1434; observed 325.1261.

#### 2.3.13 2’-Hydroxy-4-ethoxy-5’-*O*-prenylchalcone **(5M)**

Yellow solid. IR (KBr, cm^-1^): 1,646 (C=O), 1,562 (CH = CH); ^1^H-NMR (400 MHz, CDCl_3_): *δ* 1.44 (3H, d, OEt-CH_3_), *δ* 1.75 (3H, s, prenyl-CH_3_), 1.80 (3H, s, prenyl-CH_3_), 4.08 (2H, t, OEt-CH_2_), 4.56 (2H, d, J = 6.8 Hz, H-1”), 5.48 (1H, t, J = 6.8 Hz, H-2”), 6.48 (2H, m, H-4’ and H-3’), 6.92 (2H, m, H-5 and H-3), 7.45 (1H, d, J = 15.6 Hz, H-*α*), 7.59 (2H, m, H-6 and H-2), 7.81 (1H, d, J = 8.4 Hz, H-6’), 7.85 (1H, d, J = 15.6 Hz, H-*β*), and 13.57 (1H, s, chelated OH); ^13^C NMR (100 MHz, CDCl_3_): *δ* 14.70 (OEt-CH_3_), 18.22 (prenyl-CH_3_), 25.80 (prenyl-CH_3_), 63.65 (OEt-CH_2_), 65.13 (C-1”), 101.66 (C-3’), 108.14 (C-1’), 114.02 (C-4’), 114.89 (C-3 and C-5), 117.67 (C-*α*), 118.68 (C-2”), 127.33 (C-1), 130.34 (C-2 and C-6), 131.04 (C-6’), 139.13 (C-3”), 144.23 (C-*β*), 161.18 (C-4), 165.33 (C-5’), 166.53 (C-2’), and 191.80 (C=O). HR-MS for C_22_H_24_O_4_ [M + H]^+^ *m/z*: Calcd 353.1747; observed 353.2039.

#### 2.3.14 2’-Hydroxy-4-methoxy-5’-*O*-prenylchalcone **(5N)**

Yellow solid. IR (KBr, cm^-1^): 1,646 (C=O), 1,562 (CH = CH); ^1^H-NMR (400 MHz, CDCl_3_): *δ* 1.77 (3H, s, prenyl-CH_3_), 1.81 (3H, s, prenyl-CH_3_), 3.86 (3H, s, OMe), 4.51 (2H, d, J = 6.8 Hz, H-1”), 5.50 (1H, t, J = 6.8 Hz, H-2”), 6.95 (3H, m, H-3’, H-6, and H-2), 7.14 (1H, dd, J = 9.2 Hz and 3.2 Hz, H-4’), 7.40 (1H, d, J = 3.2 Hz, H-6’), 7.46 (1H, d, J = 15.6 Hz, H-*α*), 7.61 (2H, m, H-3 and H-5), 7.88 (1H, d, J = 15.2 Hz, H-*β*), and 12.62 (1H, s, chelated OH); ^13^C-NMR (100 MHz, CDCl_3_): *δ* 18.15 (prenyl-CH_3_), 25.70 (prenyl-CH_3_), 55.25 (C-OMe), 65.45 (C-1”), 108.17 (C-1’), 114.23 (C-3’), 117.48 (C-3 and C-5), 118.98 (C-2’’), 119.26 (C-4’), 124.09 (C-*α*), 125.82 (C-6’), 127.39 (C-1), 130.43 (C-2 and C-6), 138.39 (C-3’’), 145.27 (C-*β*), 150.73 (C-5’), 157.70 (C-2’), 161.91 (C-4), and 193.17 (C=O). HR-MS for C_21_H_22_O_4_ [M + H]^+^ *m/z*: Calcd 339.1590; observed 339.1392.

#### 2.3.15 2’-Hydroxy-4-dimethylamino-5’-*O*-prenylchalcone **(5O)**

Brick red solid. IR (KBr, cm^-1^): 1,646 (C=O), 1,562 (CH = CH); ^1^H-NMR (400 MHz, CDCl_3_): *δ* 1.77 (3H, s, prenyl-CH_3_), 1.81 (3H, s, prenyl-CH_3_), 3.05 (6H, s, N-CH_3_ × 2), 4.52 (2H, d, J = 6.8 Hz, H-1”), 5.51 (1H, t, J = 6.8 Hz, H-2”), 6.69 (2H, m, H-2 and H-6), 6.94 (1H, d, J = 9.2 Hz, H-3’), 7.11 (1H, dd, J = 9.2 Hz and 3.2 Hz, H-4’), 7.38 (1H, d, J = 15.2 Hz, H-*α*), 7.41 (1H, d, J = 3.2 Hz, H-6’), 7.55 (2H, m, H-3 and H-5), 7.90 (1H, d, J = 15.2 Hz, H-*β*), and 12.72 (1H, s, chelated OH); ^13^C-NMR (100 MHz, CDCl_3_): *δ* 18.22 (prenyl-CH_3_), 25.82 (prenyl-CH_3_), 40.05 (N-CH_3_ × 2), 65.72 (C-1”), 108.17 (C-1’), 111.75 (C-3’), 114.26 (C-3 and C-5), 118.88 (C-2”), 119.60 (C-4’), 120.07 (C-*α*), 122.26 (C-6’), 123.56 (C-1), 130.78 (C-2 and C-6), 138.48 (C-3”), 146.54 (C-*β*), 150.07 (C-4), 152.24 (C-5’), 157.66 (C-2’), and 193.06 (C=O). HR-MS for C_22_H_25_NO_3_ [M + H]^+^ *m/z*: Calcd 352.1907; observed 352.2721.

#### 2.3.16 2’-Hydroxy-4-benzyloxy-5’-*O*-prenylchalcone **(5P)**

Orange solid. IR (KBr, cm^-1^): 1,646 (C=O), 1,562 (CH = CH); ^1^H-NMR (400 MHz, CDCl_3_): *δ* 1.76 (3H, s, prenyl-CH_3_), 1.81 (3H, s, prenyl-CH_3_), 4.51 (2H, d, J = 6.8 Hz, H-1”), 5.12 (2H, s, benzyl-CH_2_), 5.50 (1H, t, J = 6.8 Hz, H-2”), 6.95 (1H, d, J = 9.2 Hz, H-3’), 7.02 (2H, m, H-2 and H-6), 7.14 (1H, dd, J = 9.2 Hz and 3.2 Hz, H-4’), 7.38 (6H, m, H-2’’’, H-3’’’, H-4’’’, H-5’’, H-6’’’, and H-6’), 7.46 (1H, d, J = 15.2 Hz, H-*α*), 7.61 (2H, m, H-3 and H-5), 7.88 (1H, d, J = 15.2 Hz, H-*β*), and 12.47 (1H, s, chelated OH); ^13^C-NMR (100 MHz, CDCl_3_): *δ* 18.22 (prenyl-CH_3_), 25.79 (prenyl-CH_3_), 65.21 (C-1”), 70.11 (benzyl-CH_2_), 101.67 (C-3’), 108.52 (C-4’), 113.90 (C-1’), 115.35 (C-6’’’ and C-2’’’), 117.93 (C-*α*), 118.65 (C-2”), 127.46 (C-1), 127.78 (C-4’’’), 125.82 (C-6’), 128.19 (C-6 and C-2), 128.62 (C-5 and C-3), 130.32 (C-5’’’), 131.05 (C-3’’’), 136.30 (C-4), 139.16 (C-3’’), 145.30 (C-*β*), 160.89 (C-1’’’), 165.39 (C-5’), 166.54 (C-2’), and 193.27 (C=O). HR-MS for C_27_H_26_O_4_ [M + H]^+^ *m/z*: Calcd 415.1903; observed 415.1981.

### 2.4 *In vitro* fungicidal activities

All the synthesized compounds were evaluated for *in vitro* antifungal activities through the poisoned food technique (Nene and Thapliyal, 1979) against *S. rolfsii* and *F. oxysporum*. Accordingly, *S. rolfsii* (ITCC 6474) and *F. oxysporum* (ITCC 8113) were procured form the Indian Type Culture Collection Centre (ITCC), Division of Plant Pathology, ICAR-Indian Agricultural Research Institute, New Delhi. The fungi were maintained at a temperature of 27°C for a minimum of 4–7 days on potato dextrose agar (PDA) slants to ensure viability and purity. Further subculturing was done just before the experiments. The PDA medium was prepared by thoroughly mixing 3.9 gm of PDA powder in 100 mL of distilled water to obtain a homogeneous solution before autoclaving. The autoclave conditions involved applying a pressure of 15 psi and maintaining a temperature of 121°C for 30 min. A stock solution of each compound was prepared at a concentration of 10,000 ppm by dissolving 20 mg of the respective compound in 2 mL of dimethylsulfoxide (DMSO). A series of five test concentrations (100 ppm, 50 ppm, 25 ppm, 12.5 ppm, and 6.25 ppm) was prepared by serial dilution from the stock solution. Subsequently, the test media with different concentrations were transferred into Petri dishes in quadruplicate. The Petri dishes were left undisturbed for some time to allow the compounds to diffuse evenly within the media and solidify. A sample of 1 mL DMSO served as the control. Hexaconazole 5% SC and carbendazim 50% WP were taken as the positive controls against *S. rolfsii* and *F. oxysporum*, respectively. For inoculation, a 5-mm-thick disc of the fungus comprising spores and mycelium was carefully cut from a previously subcultured fungal culture and placed at the center of a Petri plate under sterile conditions. Both the treatment and control Petri plates were placed in a biochemical oxygen demand (BOD) incubator set to a constant temperature of 25°C ± 1°C. The plates were allowed to incubate until the fungal growth in the control Petri plate reached a stage of nearly complete development. For *S. rolfsii*, this incubation period was observed to be 4–5 days, while *F. oxysporum* required a longer duration of approximately 7–10 days.

During the incubation period, regular observations were recorded for both the treatment and control Petri plates. The percentage inhibition was then calculated using Abbott’s formula (Abbott, 1925), which is a commonly used method to quantify the antifungal activity of a test compound, as follows:

$$\text{IC}=\left(I-\text{CF}\right)/\left(100-\text{CF}\right)\;x\;100,$$

where I represents percentage inhibition and CF = [(90-C)/C] × 100, with 90 being the diameter (mm) of the Petri plate and C being the growth of the fungus (mm) in the control.

The data were analyzed in quadruplicate using a web-based agricultural statistics software package (WASP 2.0). The effective dose for 50% inhibition (ED_50_) values were calculated using SPSS statistical package (version 16.0), and these values represent the concentrations of the test compounds at which 50% inhibition of fungal growth was observed.

### 2.5 Molecular docking studies

#### 2.5.1 Virtual screening and molecular docking

To determine the binding affinities and other types of interactions between the synthesized compounds with the target protein, molecular docking studies were carried out using the PyRx tool, which is a virtual screening tool that employs Vina and AutoDock 4.2. The protein–ligand binding interactions were profiled as per the procedures reported previously (Kundu et al., 2020). The target proteins of *F. oxysporum* and *S. rolfsii* were prepared first, and synthetic molecules including the control were then loaded into the PyRx virtual screening tool; the former were labeled as the macromolecules while the latter were considered the ligands. A grid box of dimensions X: 74.75, Y: 50.35, and Z: 25.0 was set around the receptor. The ligand molecules including the control and receptor molecule were docked, and the maximum exhaustiveness was computed for each ligand. All other parameters in the software application were maintained at default values. The docking results were analyzed and compared with those of the control.

Using protein sequences obtained from the NCBI GenBank and UniProt databases, the secondary structures of certain bacterial protein targets were modeled through homology. This process was aided by homologous templates found in the NCBI and RCSB PDB Protein Data Bank databases. The molecular and biological features of the query sequences were searched and annotated using the BLAST servers available at http://blast.ncbi.nlm.nih.gov. Using the NCBI Blast tool, the PDB database was searched for templates that could be used to model the secondary structures of the query sequences. For the three-dimensional structural modeling of each target protein, Modeler software (version 9.24) was utilized. ProSA-Web was used to compute an all-encompassing quality score that can be employed to verify whether the z-score of the input structure is within the range of scores commonly observed for native proteins of comparable sizes. To evaluate the stereochemical accuracy of the modeled protein, thorough analysis was conducted using PROCHECK-Saves. The Ramachandran plot was used to display the phi-psi torsion angles for all residues in the ensemble.

The ICM Pocket Finder is a powerful tool that can accurately detect and locate ligand binding sites within protein, DNA, or RNA structures. The identification of protein–ligand binding sites involves analyzing the grid potential maps of the van der Waals interactions between the receptors and surfaces, which are then contoured. The physical properties of the pockets are calculated and tabulated to obtain a drug-likeness score. Various factors can impact the binding of ligands to a pocket, such as the size and shape of the pocket, its level of exposure, the presence of hydrophobic regions, and the overall compactness of the pocket. All these properties are determined using ICM Pocket Finder (Sheridan et al., 2010). The MolSoft ICM Pocket Finder algorithm uses a method to measure the “drugability” of a protein target using a metric called the drug-like density (DLID).

#### 2.5.2 Analyses and visualization of docking results

Analyses and visualization of the docking results were conducted to examine and interpret the docking interactions. The selection of docked poses for the protein–ligand complexes was based on a combination of factors, including the docking energy profile and manual analysis of the interaction patterns with active site residues. The docked conformations and their corresponding interaction patterns were visualized and assessed using PyMOL and the Discovery Studio platform (DeLano, 2002; Discovery Studio Visualization, 2016).

## 3 Results and discussion

### 3.1 Synthesis and characterization

**FIGURE 3:**
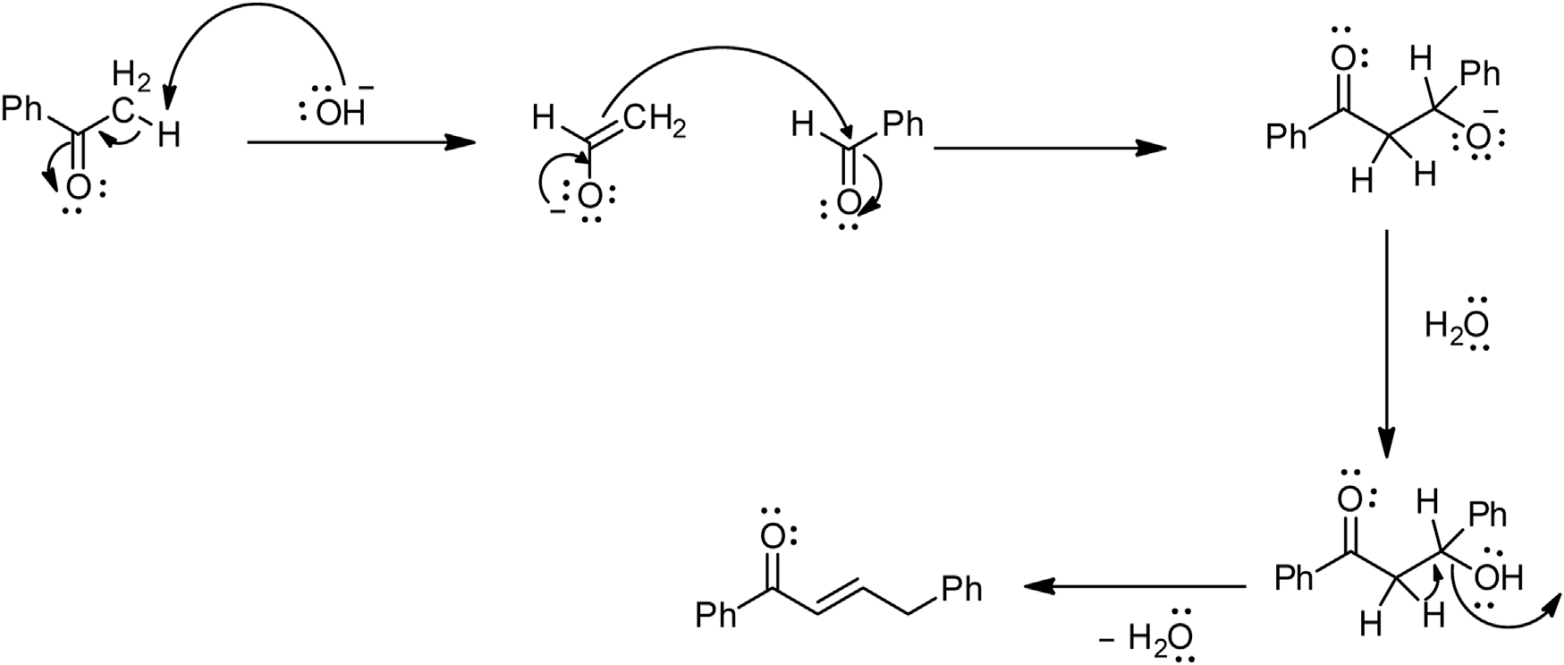
Mechanism of Claisen–Schmidt condensation.

Among the most used synthetic routes for chalcones (Farooq and Ngaini, 2019), the main route is Claisen–Schmidt condensation (Figure 3), which involves condensation between a benzaldehyde and an acetophenone (Guida et al., 1997; Nasir Abbas Bukhari et al., 2013). This reaction takes place in polar solvents at temperatures ranging from 50°C to 100°C and often requires several hours (Rammohan et al., 2020).

In the present study, prenylated chalcones were synthesized using both the conventional and microwave-assisted methods. The yields were higher in the microwave-assisted method (86%–92%) than the CM (71%–83%), and the reaction time was considerably lowered to 1–4 min compared to that of the CM that ranges between 12 h and 48 h (Table 2). In the ^1^H-NMR spectra of all the synthesized compounds, doublets were observed at *δ* ranges of 7.01–7.80 (H-*α*) and 7.7–8.9 (H-*β*) with J values of 15–16 Hz, which is a characteristic peak of the olefinic bond of the chalcone moiety, clearly indicating the condensation of prenylated acetophenone with different benzaldehydes. In the ^13^C-NMR spectra, peaks were observed at *δ* ranges of 120–130 (C-*α*) and 137.2–141.3 (C-*β*) for HC = CH and *δ* range of 190–195 for C=O in all the synthesized compounds.

**TABLE 2:** Comparisons of reaction time and yield (%) of the microwave and conventional methods.

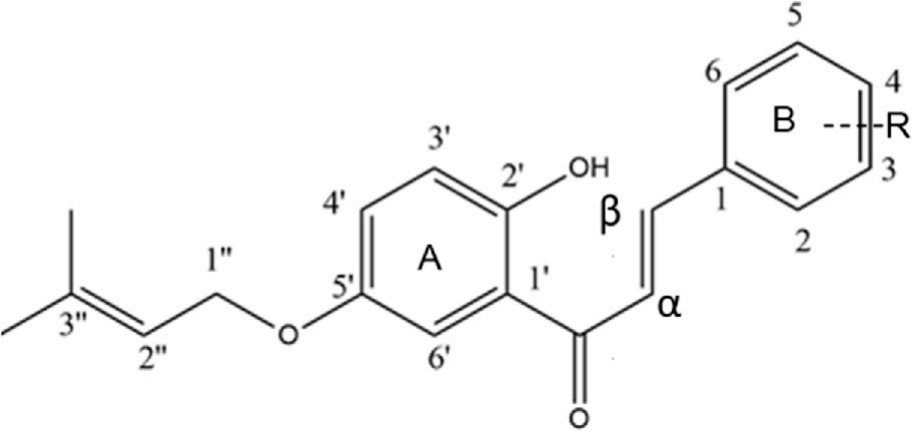						
S. no.	Compound	R	Conventional method		Microwave method	
			Time (h)	Yield (%)	Time (min)	Yield (%)
1	**5A**	3-NO_2_	24	81.22	3.2	90.40
2	**5B**	2,6-Cl	16	83.60	1.5	90.66
3	**5C**	2,4-Cl	18	82.54	1.5	89.48
4	**5D**	3-Cl	20	80.90	1.5	89.30
5	**5E**	4-Br	22	79.56	1.5	90.56
6	**5F**	3-Br	24	78.80	1.5	89.44
7	**5G**	2-Br	24	80.40	1.5	91.60
8	**5H**	4-NO_2_	22	71.22	3.2	86.88
9	**5I**	3,4,5-OMe	18	79.20	1.5	89.80
10	**5J**	2-NO_2_	24	75.50	3.2	88.28
11	**5K**	4-OH	48	77.46	2.4	90.80
12	**5L**	3-OH	48	78.30	2.4	91.40
13	**5M**	4-OEt	22	80.22	1.5	91.60
14	**5N**	4-OMe	24	81.30	1.5	90.68
15	**5O**	4-N(CH_3_)_2_	24	79.40	3.2	89.30
16	**5P**	4-OCH_2_Ph	22	80.56	3.2	90.22

Bold values represent the codes for compounds.

### 3.2 Detailed 1D and 2D NMR spectra of compound **5A**

The compound **5A** was obtained as an orange solid from methanol with a yield of 90%. The absorption at 1,677 cm^-1^ in the IR spectrum showed the presence of the carbonyl group in the molecule; this was supported by a signal at *δ* = 190.82 for the carbonyl in the ^13^C-NMR spectrum. The ^1^H-NMR spectrum of **5A** showed two separate singlets integrating for six protons at *δ* = 1.73 and 1.80 for the two vinylic methyl groups. A doublet was observed at *δ* = 4.54 (J = 6.8 Hz) from integrating two protons due to H-1”, and a triplet was seen at *δ* = 5.49 (J = 6.8 Hz) for H-2”. A doublet integrating for one proton was seen at *δ* = 6.97 with J = 9.2 Hz due to H-3’. A double doublet was seen at *δ* = 7.08 from integrating one proton with J values of 9.2 Hz and 3.2 Hz for H-4’. A doublet was obtained at *δ* = 7.33 from integrating one proton with J = 3.2 Hz due to H-6’, and a triplet was seen at *δ* = 7.57 from integrating one proton with J = 8 Hz due to H-5. Two doublets were observed at *δ* = 7.67 and 7.75 from integrating one proton each with J = 16 Hz due to H-*α* and H-*β*, respectively; the presence of these two protons H-*α* and H-*β* confirms the prevalent chalcone moiety in the structure. Two multiplets were observed at *δ* = 8.22 and 8.44 from integrating one proton each for H-4 and H-2, respectively. The chelated OH showed a singlet at *δ* = 12.18.

In the ^13^C-NMR the spectrum, two methylene carbons were seen in the prenyl chain at *δ* = 18.21 and 25.75, respectively. The C-1” to which the carbonyl moiety of acetophenone was attached was seen at *δ* = 65.38. The signal due to C-3’ and C-4’ was found at *δ* = 115.09 and that for C-2” of the prenyl side chain was seen at *δ* = 119.11. The signal for C-1’ was obtained at *δ* = 119.44. In the aromatic ring B, carbons 6, 4, and 2 were seen at *δ* = 121.52, 122.21, and 124.14, respectively. The *α* and *β* carbons of the olefinic double bond were found at *δ* = 129.85 and 148.66, respectively. The signal at *δ* = 129.85 was also attributed to C-5 and C-6’. The aromatic carbons 1 and 3 of ring B were found at *δ* = 134.08 and 137.29, respectively. The signal at *δ* = 138.68 was due to the C-3” of the prenyl moiety. The carbon (C-5’) in ring A to which the prenyl moiety was attached through O showed a signal at *δ* = 152.65. The C-2’ in ring A to which a hydroxyl group was attached showed a signal at *δ* = 152.98. The nature of these carbons was confirmed further with the help of DEPT NMR spectroscopy, in which DEPT 45 showed signals for carbons 2, 4, 5, 6, 3’, 4’, 6’, 1’’, 2’’, *α*, *β*, and prenyl-CH_3_. DEPT 90 showed signals for carbons 2, 4, 5, 6, 3’, 4’, 6’, 2’’, *α*, and *β*. DEPT 135 showed only one negative signal for C-1”, indicating the CH_2_ nature, and all other carbons were found at positive values.

**FIGURE 4:**
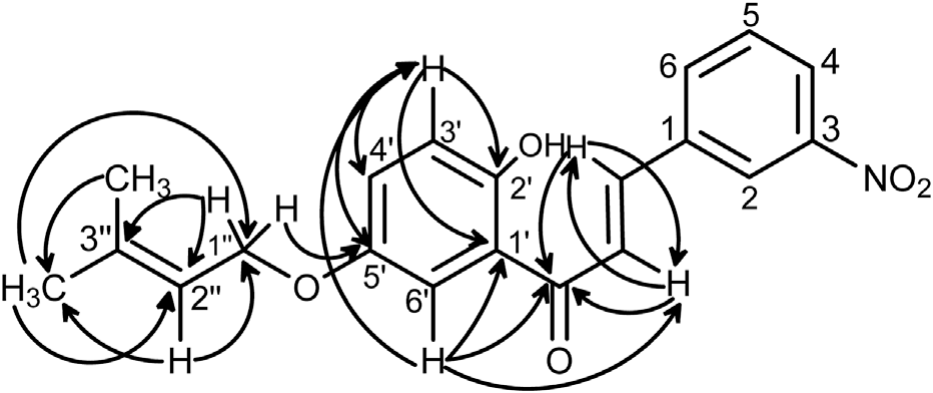
Two-dimensional NMR spectroscopy as well as HMBC and COSY correlations for compound **5A**.

The above structure was confirmed with the help of two-dimensional (2D) NMR spectroscopy of the compound (Figure 4). The signal at *δ* = 4.54 assigned to H-1” showed coupling with the carbon at *δ* = 152.65 in its HMBC spectrum, which is C-5’; it also showed significant coupling with the carbons at *δ* = 119.11 (C-2”) and 138.68 (C-3”). The signal at *δ* = 5.49 assigned to H-2” showed correlations with the CH_3_ carbon of the prenyl moiety and carbon 1” at *δ* = 65.38. The proton at *δ* = 6.97 attached to the carbon at 3’ showed correlations with the carbons at 1’, 2’, 4’, and 5’. The *α* and *β* carbons showed coupling with the carbonyl carbon at *δ* = 190.82. Furthermore, the proton at *δ* = 7.33 that is attached to C-6’ showed correlations with the carbonyl carbon at *δ* = 190.82 and C-1’ at *δ* = 119.44. In COSY, the proton attached to C-6’ showed a correlation with the proton attached to C-*α*. Moreover, the two *trans* protons attached at the *α* and *β* carbons showed correlations in COSY. The prenyl-CH_3_ protons at *δ* = 1.73 and 1.80 showed correlations with the protons attached at C-1” and C-2”. The proton attached to the carbon at *δ* = 129.85, which is C-6’, showed coupling with the proton attached to C-3’ at *δ* = 115.09.

### 3.3 Comparative assessments of the conventional and microwave methods

Microwave synthesis aligns perfectly with the principles of green chemistry as it employs recyclable organic solvents in the synthesis process, prevents wastage, and lowers energy consumption (Sahoo et al., 2023). To assess the greenness of the method used in this study, comparisons were made between the conventional and microwave methods (Table 3) using green chemistry matrices by considering 2’-hydroxy-4-hydroxy-5’-*O*-prenylchalcone (**5K**) as an example. For the MM, there was a 13.34% increase in yield and 1,200 times reduction in the heating time than the CM; the energy consumption also reduced by 411.9–388.7 times due to the reduced heating time. Environmental factor (EF) values showed that there was a 95% reduction in the production of wastes for producing 1 kg of the compound. The MM was also found to be more carbon efficient by 17.7% and reaction mass efficient by 29.6%. In conclusion, the utilization of the MM for synthesizing prenylated chalcones demonstrates remarkable improvements over the CM.

**TABLE 3:** Comparative assessment of the conventional and microwave methods based on various green chemistry matrices.

S. no.	Matrix	Conventional method	Microwave method	Improvement
1	Overall yield (%)	77.46	90.80	13.34% increase
2	Heating time (s)	48 h	144 s	1,200 times shorter
3	Energy consumption (kWh)	27.6	0.067–0.071	411.9–388.7 times lower
4	Environmental factor	4.0	0.2	95% reduction
5	Carbon efficiency (%)	34.1	51.8	17.7% increase
6	Reaction mass efficiency (%)	34.2	63.8	29.6% increase

### 3.4 *In vitro* fungicidal activity

**FIGURE 5:**
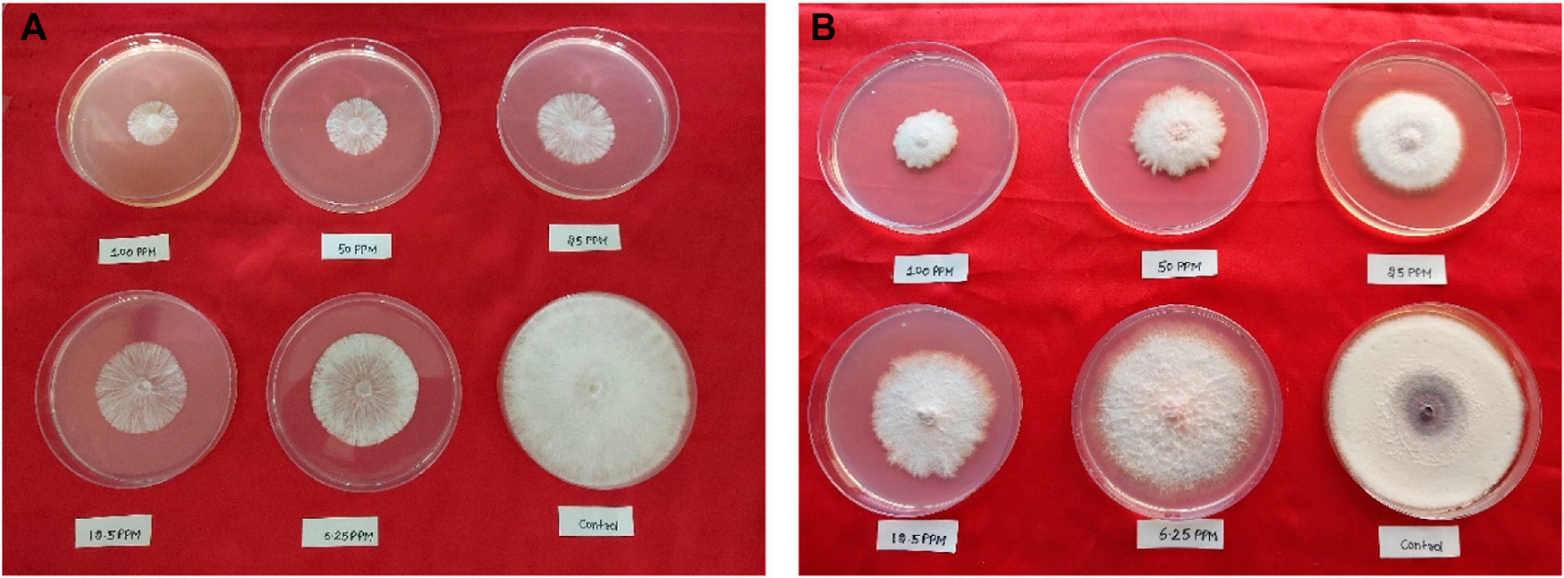
*In vitro* antifungal activities of compound **5P** against **(A)** *S. rolfsii* and **(B)** *F. oxysporum.*

All the synthesized compounds showed significant antifungal activities (Table 4, 5). Control experiments were conducted in a manner consistent with the experimental conditions but without the inclusion of the synthesized compounds. Hexaconazole 5% SC and Carbendazim 50% WP served as the standards for comparing the antifungal activities. The compound 2’-hydroxy-4-benzyloxy-5’-*O*-prenylchalcone (**5P**) possessed the highest activities against both *S. rolfsii* and *F. oxysporum*, with ED_50_ of 25.02 and 31.87 mg/L, respectively. The corresponding ED_50_ values of the commercial fungicides were 8.57 mg/L (Hexaconazole 5% SC) and 9.01 mg/L (Carbendazim 50% WP) (Figure 5). The *in silico* technique of SwissADME was performed to better understand the pharmacokinetic profile of the compound **5P**, as described in Table 6 (Daina et al., 2017).

**TABLE 4:** *In vitro* antifungal activities of the synthesized compounds against *S. rolfsii*.

Compound	ED_50_ (ppm)^a^	Lower fiducial limit	Upper fiducial limit	X^2^	Regression equation
**5A**	43.24^abcd^	33.21	60.64	0.338	1.12x + -1.84
**5B**	35.68^def^	28.29	46.69	1.592	1.20x + -1.90
**5C**	38.52^cdef^	29.24	54.27	1.688	1.12x + -1.84
**5D**	46.86^cdef^	36.05	66.06	0.710	1.20x + -1.90
**5E**	44.20^a^	32.28	68.04	0.141	0.96x + -1.52
**5F**	44.65^def^	34.45	62.35	0.344	1.20x + -1.90
**5G**	30.28^efg^	23.68	39.59	1.519	1.12x + -1.64
**5H**	52.56^cde^	40.60	74.19	0.353	1.12x + -1.84
**5I**	49.19^abc^	38.12	68.70	0.840	1.12x + -1.84
**5J**	48.53^fgh^	36.38	71.93	0.353	1.12x + -1.84
**5K**	40.07^abcd^	29.67	59.20	0.301	0.96x + -1.52
**5L**	36.16^abcd^	27.67	49.89	0.614	1.20x + -1.90
**5M**	25.24^cdef^	18.22	35.03	0.216	0.96x + -1.32
**5N**	32.42^efg^	24.82	43.99	0.784	1.12x + -1.84
**5O**	51.84^def^	39.36	75.43	0.762	0.96x + -1.52
**5P**	25.02^gh^	19.89	31.46	0.721	1.60x + -2.20

^a^ No significant differences in mean for the same letters; [CD (0.01) = 0.655] hexaconazole 5% SC; ED_50_ = 8.57 ppm.

**TABLE 5:** *In vitro* antifungal activities of the synthesized compounds against *F. oxysporum*.

Compound	ED_50_ (ppm)^a^	Lower fiducial limit	Upper fiducial limit	X^2^	Regression equation
**5A**	45.41^fg^	32.85	71.53	0.224	0.96x + -1.52
**5B**	33.03^h^	24.78	46.20	0.405	1.00x + -1.50
**5C**	42.66^gh^	32.26	61.38	0.298	1.12x + -1.84
**5D**	40.17^g^	29.39	60.67	0.634	0.96x + -1.52
**5E**	57.42^cd^	40.92	96.16	1.560	0.96x + -1.72
**5F**	35.60^g^	27.02	49.60	1.939	1.12x + -1.84
**5G**	61.12^cde^	46.06	91.18	0.492	1.12x + -2.15
**5H**	60.58^ef^	44.03	97.32	0.854	1.12x + -1.84
**5I**	60.00^c^	44.88	90.77	1.643	1.12x + -2.04
**5J**	75.47^b^	53.96	126.66	0.470	1.12x + -2.04
**5K**	55.52^cd^	40.24	89.15	0.590	0.96x + -1.72
**5L**	47.35^def^	34.73	73.01	0.954	1.12x + -1.84
**5M**	46.36^fg^	36.00	64.25	0.509	1.28x + -2.16
**5N**	56.94^cd^	41.50	90.58	0.155	0.96x + -1.72
**5O**	49.46^de^	36.86	74.30	0.708	1.00x + -1.75
**5P**	31.87^i^	25.04	41.71	0.467	1.12x + -1.80

^a^ No significant differences in mean for the same letters; [CD (0.01) = 0.281] carbendazim 50% WP; ED_50_ = 9.01 ppm.

**TABLE 6:** Physicochemical, pharmacokinetic, and drug-like properties of compound **5P**.

Compound 5P 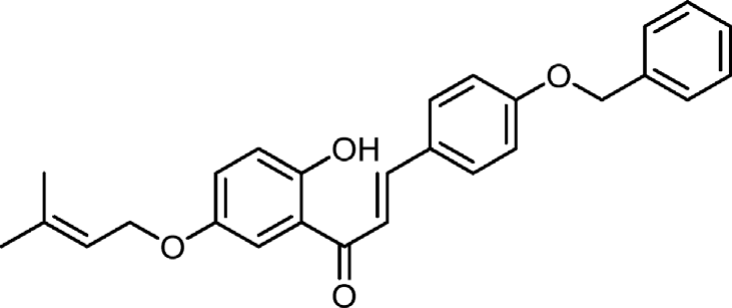	
Physiological property	
Formula	C_27_H_26_O_4_
Molecular weight (g/mol)	414.4
Number of heavy atoms	31
Number of rotatable bonds	9
Number of hydrogen bond acceptors	4
Number of hydrogen bond donors	1
Pharmacokinetic parameter	
Log *P* o/w (MLOGP)	3.96
Gastrointestinal absorption	High
Blood–brain barrier permeation	No
Drug-like properties	
Lipinski rule	Yes; 0
	Violation
Bioavailability	0.55

### 3.5 SAR studies

**FIGURE 6:**
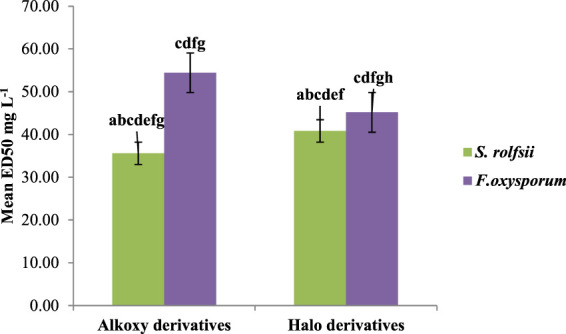
Mean ED_50_ values for alkoxy and halo derivatives against *S. rolfsii* and *F. oxysporum.*

**FIGURE 7:**
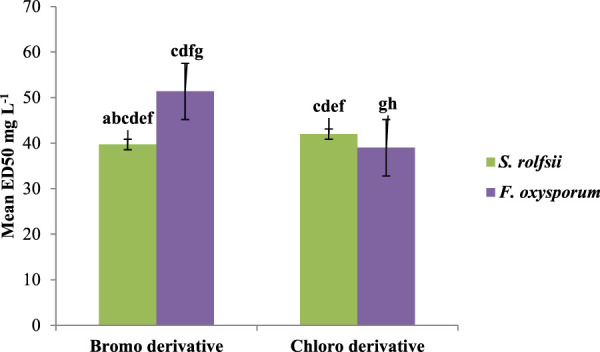
Effects of bromo and chloro groups on antifungal activities against *S. rolfsii* and *F. oxysporum*.

A series of prenylated chalcones was synthesized, and their structural properties were examined to investigate their potential as antifungal agents. Both fungi responded differently to changes in electron density in the test compounds. The alkoxy substitution on ring B with a mean ED_50_ value of 35.62 mg/L against *S. rolfsii* was found to be more effective than halo substitution with a mean ED_50_ value of 40.84 mg/L. On the other hand, for *F. oxysporum*, the halo derivative with a mean ED_50_ 45.16 mg/L was found to be more effective (Figure 6). Among halo derivatives, bromo compounds (mean ED_50_ 39.71 mg/L) were more effective against *S. rolfsii* while chloro compounds (mean ED_50_ 39.01 mg/L) worked better against *F. oxysporum* (Figure 7).

**FIGURE 8:**
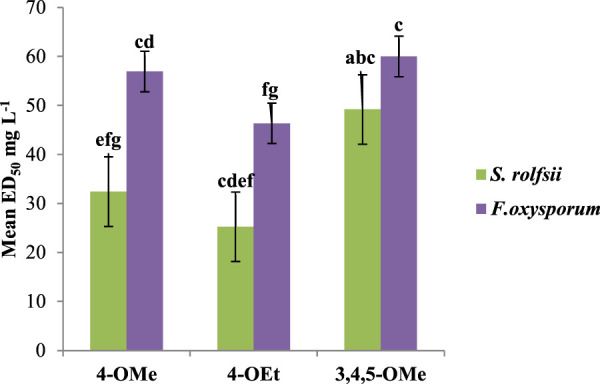
Effects of the number of substituents among the alkoxy derivatives on ED_50_ values for antifungal activity.

The number of substituents on ring B had an impact on the antifungal activity. For the alkoxy derivatives that donate electrons, the antifungal activity decreased as the number of substituents increased. The compound with one methoxy (-OCH_3_) group was found to be a more potent antifungal agent than that with three methoxy groups against both *S. rolfsii* (ED_50_ 32.42 mg/L) and *F. oxysporum* (ED_50_ 56.94 mg/L). The introduction of three methoxy groups resulted in decreased activities against both *S. rolfsii* (ED_50_ 49.19 mg/L) and *F. oxysporum* (ED_50_ 60.00 mg/L). On substituting the methoxy group with the ethoxy group (-OCH_2_CH_3_), the activities increased substantially against both *S. rolfsii* (ED_50_ 25.24 mg/L) and *F. oxysporum* (ED_50_ 46.36 mg/L). This increase in activity upon increasing the chain length may be attributable to the increased hydrophobicity of the molecule (Figure 8).

**FIGURE 9:**
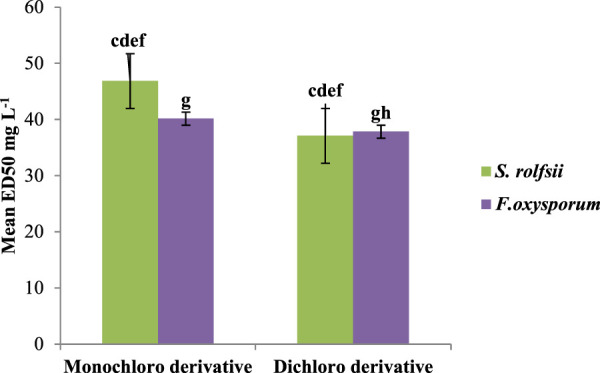
Comparative analysis of the influence of number of substituents on antifungal activity.

Upon going from monochloro substitution to dichloro substitution, the mean ED_50_ values decreased, resulting in increased activities against both *S. rolfsii* (mean ED_50_ 37.10 mg/L) and *F. oxysporum* (mean ED_50_ 37.85 mg/L). When attached to carbon atoms, chlorine atoms are not readily polarizable, leading to increased hydrophobicity and lipophilicity of the compound and causing enhanced penetration of the membranes. The presence of more chlorine substituents on the benzaldehyde ring could help avoid intermolecular interactions, causing an increase in activity (Figure 9).

**FIGURE 10:**
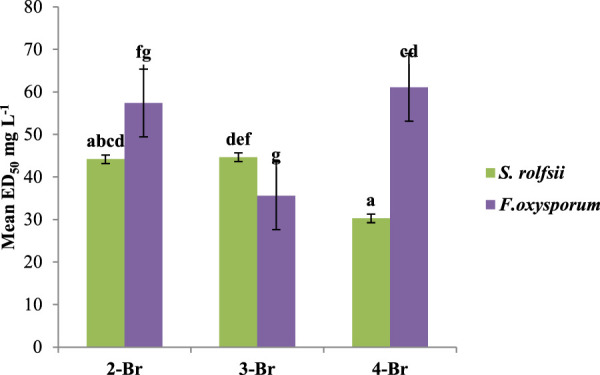
Effects of various positions of the bromine atom in the benzaldehyde ring on the antifungal activities among bromo derivatives.

The positions of the atoms also had different effects on the antifungal properties. For instance, compounds with 2,6-dichloro substitutions showed higher activities against both fungi compared to those with 2,4-dichloro substitutions. The observed higher activities of the compounds with 2,6-dichloro substitutions could be attributed to the specific spatial arrangements and interactions within the binding sites of the target proteins. The placement of the two chlorine atoms at 2 and 6 positions may have led to a more favorable fit within the binding pocket of target protein, hence enhancing the binding affinity. Among the bromo compounds, 4-Br (ED_50_ 30.28 mg/L) was found to be the most effective against *S. rolfsii*, while 3-Br was the most effective against *F. oxysporum* (ED_50_ 35.60 mg/L) (Figure 10).

**FIGURE 11:**
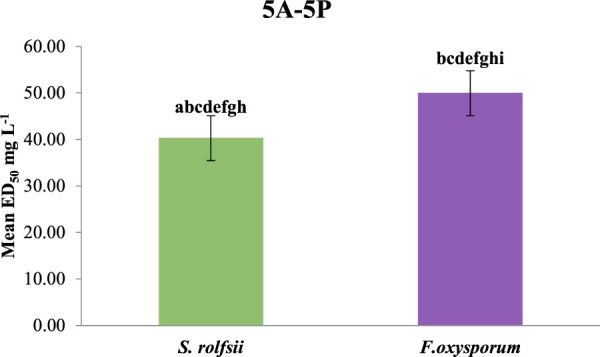
Comparative effects of all the synthesized compounds (**5A-5P**) on antifungal activities against *S. rolfsii* and *F. oxysporum*.

The compounds having benzyloxy substituents on ring B were found to be the most active molecules against both fungi, with the lowest ED_50_ values of 25.02 mg/L (against *S. rolfsii*) and 31.87 mg/L (against *F. oxysporum*). Other substituents introduced into ring B, like N,N-dimethylethanamine or nitro and hydroxyl groups, showed mild-to-moderate antifungal activities. Finally, upon comparing the mean ED_50_ values of all the synthesized compounds against both fungi, it was found that the compounds were generally more effective against *S. rolfsii* (mean ED_50_ 40.28 mg/L) than *F. oxysporum* (mean ED_50_ 49.94 mg/L) (Figure 11).

### 3.6 Molecular docking

Molecular docking is a powerful computational tool that helps to understand how proteins interact with different molecules. Nowadays, molecular docking plays an important role in the development of new agrochemicals by helping in the design, optimization, and understanding of how these chemicals interact with their target proteins in plants and pests. In this study, molecular docking was used to assess two fungi, namely, *S. rolfsii* with focus on its EF1*α* and RPB2 gene sequences and *F. oxysporum* with focus on its FoCut5a sequence as the receptors, and obtain insights into the interactions between each synthesized compound and specific binding sites. The EF1*α* and RPB2 genes are essential for the growth and survival of *S. rolfsii*; hence, compounds inhibiting these targets can act as potential antifungal agents by disrupting their native biofunctional activities. Similarly, the FoCut5a enzyme helps *F. oxysporum* to break the cutin-assisted barrier in plants, facilitating its primary infection. Therefore, this enzyme can act as a potential target for the development of new antifungal agents against *F. oxysporum*. Molecular docking was performed for the synthesized compounds against *S. rolfsii* and *F. oxysporum*. All compounds showed promising interactions with the binding sites. The corresponding binding affinities were calculated based on hydrogen dehydration (HYDE) scoring. The other important factors of molecular docking studies, i.e., octanol−water partition coefficient (Log P), ligand efficiency (LE), and lipophilic ligand efficiency (LLE), were calculated, as listed in Tables 7 and 8. The molecular docking studies suggest that the required binding energies of the docked molecules range from −38.3538 to −26.6837 kcal/mol for *S. rolfsii* and −43.400 to −23.839 kcal/mol for *F. oxysporum*. The other parameters also showed that the compounds form stable complexes with the protein molecules.

**TABLE 7:** Binding energy affinities of the synthesized compounds against *S. rolfsii.*

Synthesized compound	Log P	Binding affinity range (aM)	^a^Binding energy	^b^Ligand efficiency	^c^Lipophilic ligand efficiency
**5P**	6.4256	3.17<KI<315.62	−38.3538	0	--
**5M**	5.032	16.15<KI<1,605.05	−38.1437	0	--
**5G**	4.5415	17.10<KI<1,699.58	−37.8504	0	-
**5N**	4.6993	19.14<KI<1902.03	−37.9695	0	-
**5B**	5.9401	21.08<KI<2094.44	−37.589	0	--
**5L**	4.6419	29.17<KI<2899.06	−36.678	0	--
**5C**	5.2867	38.60<KI<3835.84	−36.0256	0	--
**5K**	4.3389	40.99<KI<4072.85	−35.9506	0	-
**5A**	4.6591	43.00<KI<4272.97	−31.0587	-	--
**5E**	5.3958	58.83<KI<5846.07	−35.0657	0	--
**5J**	4.3389	98.89<KI<9825.84	−33.4835	0	--
**5F**	5.3958	244.76<KI<24318.50	−31.4308	0	--
**5I**	4.5415	1528.86<KI<151901.62	−26.6837	-	--

**TABLE 8:** Binding energy affinities of the synthesized compounds against *F. oxysporum.*

Synthesized compound	LogP	Binding affinity range (aM)	^a^Binding energy	^b^Ligand efficiency	^c^Lipophilic ligand efficiency
**5P**	6.4256	2.01<KI<199.95	−43.400	0	--
**5B**	5.9401	2.73<KI<271.30	−43.172	-	--
**5F**	5.3958	9.56<KI<950.53	−40.073	0	--
**5D**	5.2867	24.46<KI<2430.76	−37.746	0	--
**5C**	5.9401	30.28<KI<3008.89	−37.217	0	--
**5A**	5.3958	36.33<KI<3610.08	−36.765	0	--
**5M**	5.032	63.30<KI<6289.77	−35.359	0	--
**5L**	4.3389	169.56<KI<16847.23	−32.947	0	--
**5O**	4.6993	235.56<KI<23404.36	−32.132	-	--
**5K**	4.3389	324.66<KI<32257.42	−31.336	0	--
**5N**	4.6419	407.73<KI<40510.45	−30.772	-	--
**5I**	4.5415	626.78<KI<62274.83	−24.471	-	--
**5E**	5.3958	701.59<KI<69707.41	−29.426	-	--
**5H**	4.5415	2883.20<KI<286463.91	−25.517	-	--
**5J**	4.5415	6680.89<KI<663786.44	−23.839	-	--

**FIGURE 12:**
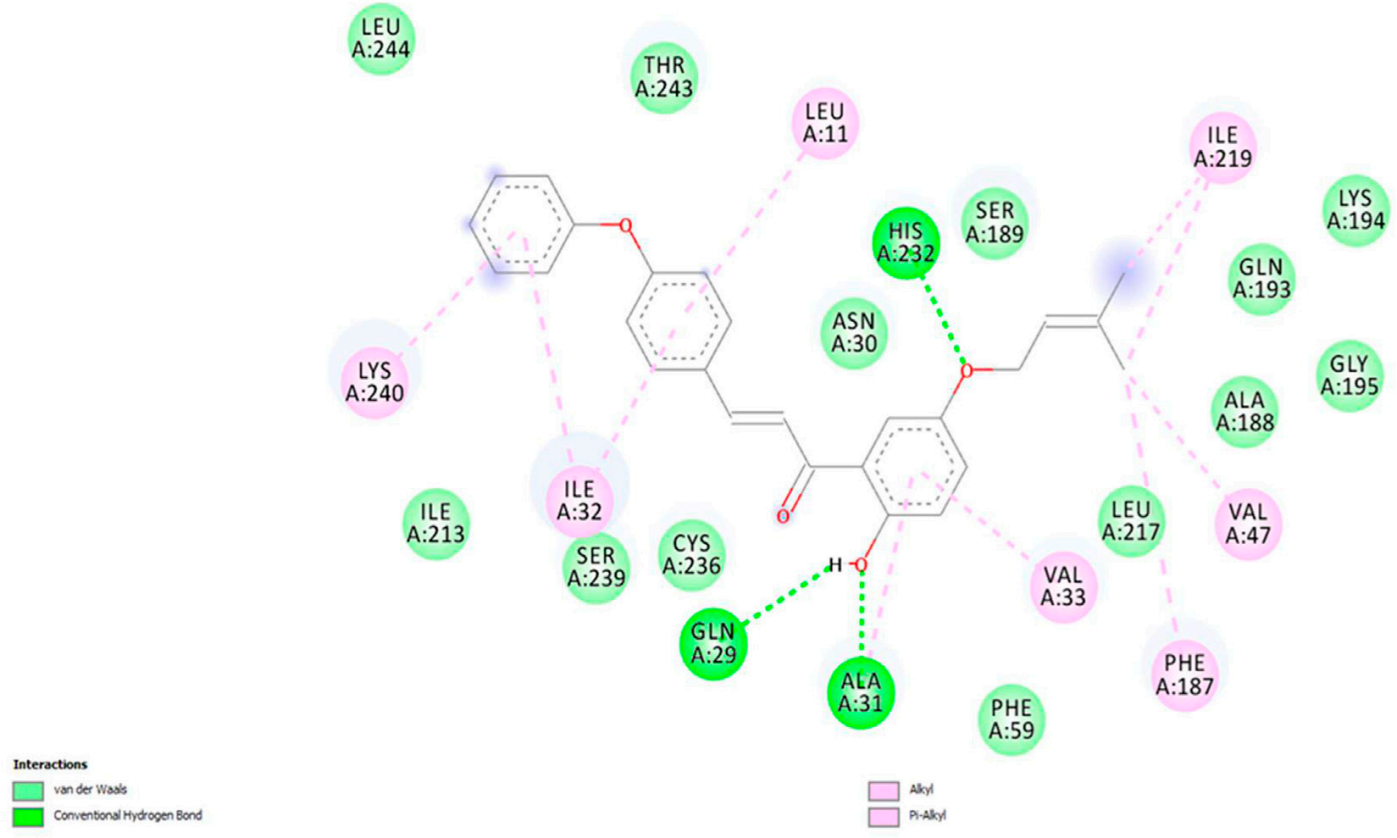
2D image of compound **5P** showing various interactions with different amino acids.

**FIGURE 13:**
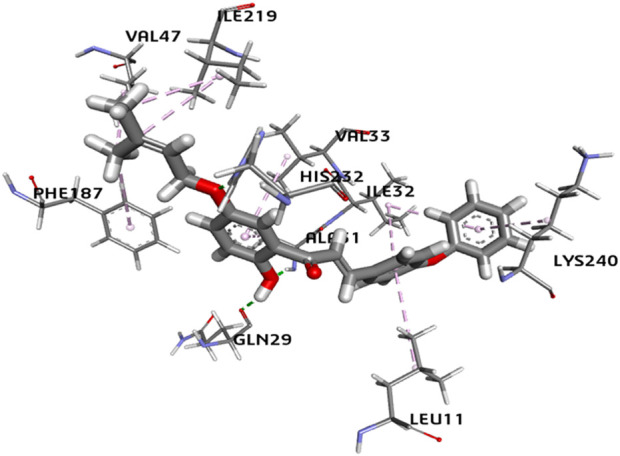
3D image of compound **5P** showing the predicted spatial arrangement of the compound within the binding site of the target fungus *S. rolfsii*.

The binding interactions of the most potent compound, **5P**, against *S. rolfsii* are explained in terms of the conventional H bonds, van der Waals bonds, and C-H bonds among the ligand–protein complex. Different interactions are shown in the 2D and 3D images (Figures 12, 13), suggesting favorable positive bindings between the molecule and specific amino acid residues. Compound **5P** showed the highest binding affinity of −38.3538 kcal/mol among all the synthesized compounds. As evident from the images, the prenyloxy side chain showed favorable steric interactions with HIS A: 232, ILE A: 219, SER A: 189, LYS A: 194, GLN: 193, GLY A: 195, ALA A: 188, LEU A: 217, VAL A: 47, and PHE A: 187, while the side chain of ring B was found to engage in interactions with ILE A: 32, ILE A: 213, LEU A: 11, SER A: 239, CYS A: 236, and THR A: 243 residues. In addition, ring A appeared to be involved in interactions with GLN A: 29, ALA A: 31, VAL A: 33, and PHE A: 59. All other compounds showed very high binding energies and favorable interactions with the target molecules.

**FIGURE 14:**
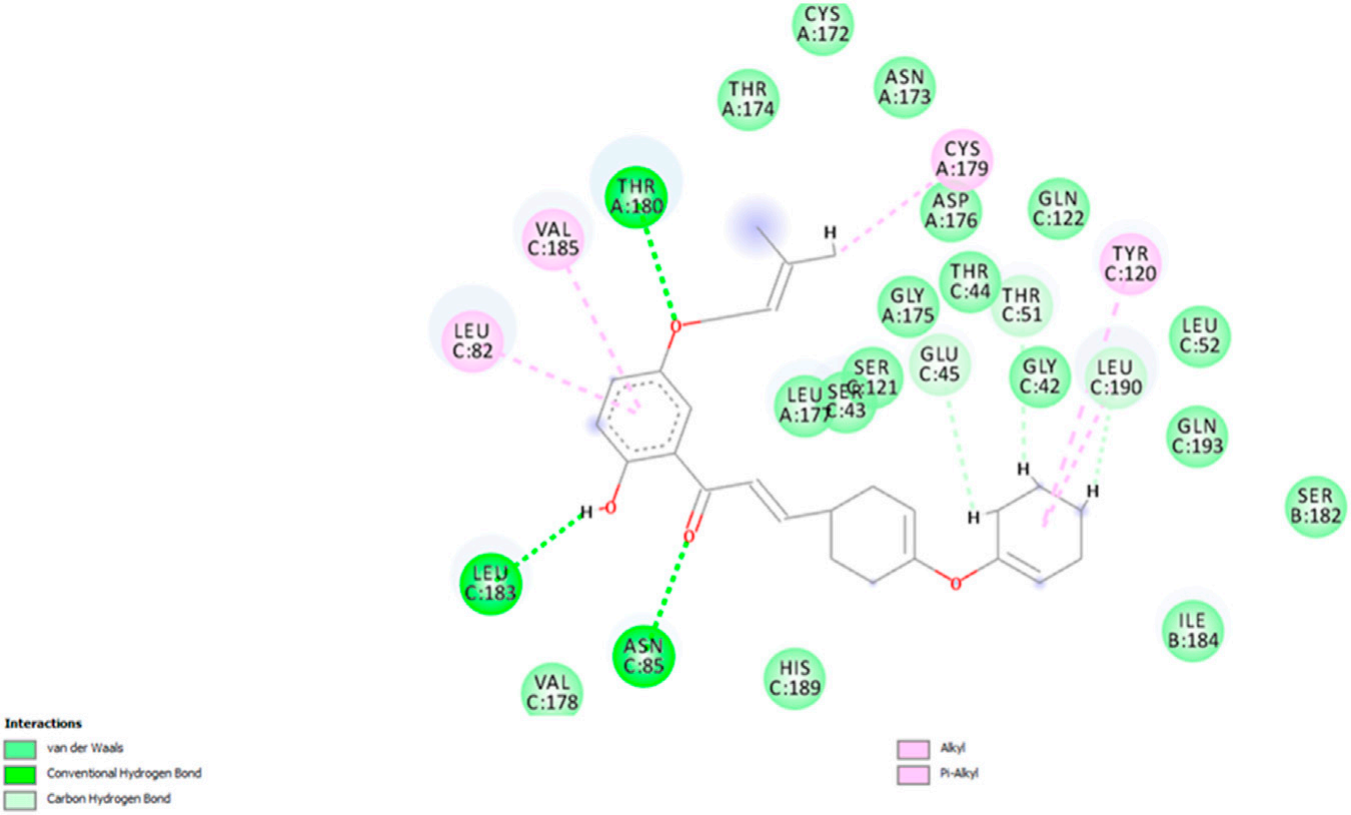
2D image of compound **5P** showing various interactions with different amino acids.

**FIGURE 15:**
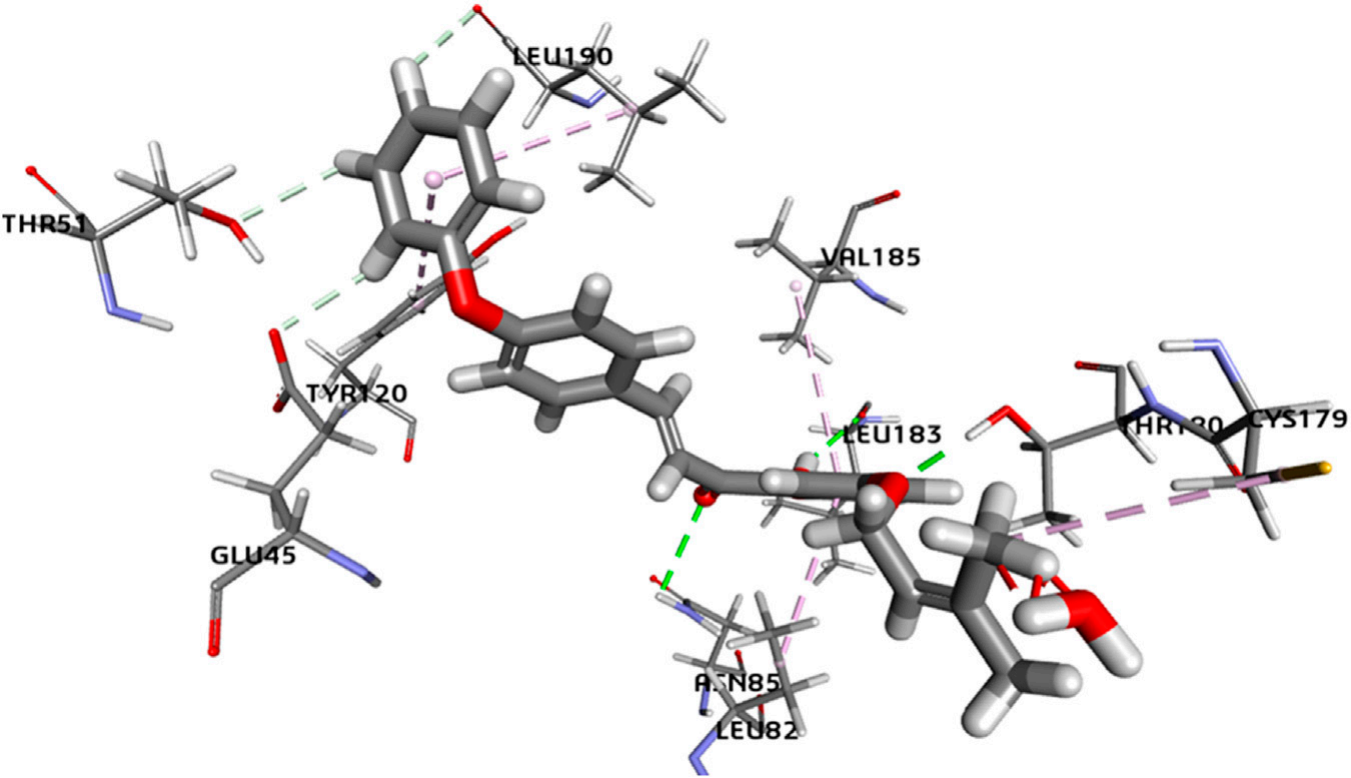
3D image of compound **5P** showing the predicted spatial arrangement of the compound within the binding site of the target fungus *F. oxysporum*.

In the case of *F. oxysporum*, the most potent compound based on *in vitro* studies was **5P**, and its 2D and 3D images showing various interactions are shown in Figures 14 and 15. Compound **5P** showed the highest binding affinity of −43.400 kcal/mol. It was seen that the prenyloxy side chain showed favorable steric interactions with THR A: 180, CYS A: 179, LEU A: 177, SER C: 43, GLY A: 175, THR C: 44, GLN C: 122, SER C: 121, THR A: 174, and ASP A: 176 residues, while ring B was found to engage in interactions with LEU C:190, LEU C: 52, TYR C: 120, ILE B: 184, GLY C: 42, GLU C: 45, THR C: 51, SER B: 182, GLN C: 193, and GLN C: 193 residues. Moreover, ring A was found to be involved in interactions in LEU C: 82, VAL C: 185, and LEU C: 183. All other compounds showed very high binding energies, and these docking results are in good agreement with the observations of the *in vitro* studies.

## 4 Conclusion

A series of 16 prenylated chalcone derivatives (**5A-5P**) was synthesized using the microwave-assisted green method and characterized by different spectroscopic techniques. All synthesized compounds were found to be novel. Out of the synthesized compounds, 2’-hydroxy-4-benzyloxy-5’-*O*-prenylchalcone (**5P**) possessed the highest activities against both *S. rolfsii* and *F. oxysporum*, with ED_50_ values of 25.02 mg/L and 31.87 mg/L, respectively. The *in vitro* results were supported by detailed SAR studies. Molecular docking studies showed the biological importance of these synthesized compounds, with the binding energies of the docked molecules for *S. rolfsii* ranging from −38.3538 to −26.6837 kcal/mol and those for *F. oxysporum* ranging from −43.400 to −23.839 kcal/mol. Other docking parameters were also evaluated, which showed that the synthesized compounds formed stable complexes with protein molecules. Thus, it is concluded that the synthesized prenylated chalcones hold immense potential and can be explored further for new antifungal agents.

## Data Availability

The original contributions presented in the study are included in the article/Supplementary Material; further inquiries may be directed to the corresponding author.

## References

[B1] Abbott W. S. (1925). A method of computing the effectiveness of an insecticide. J. Econ. Entomol. 18, 265–267. 10.1093/jee/18.2.265a

[B2] Agrios G. N. (2004). Losses caused by plant diseases. Plant Pathology. Oxford, UK: Elsevier, 29–45.

[B3] Alawiye T. T. Babalola O. O. (2021). Metabolomics: current application and prospects in crop production. Biologia 76 (1), 227–239. 10.2478/s11756-020-00574-z

[B4] Binate G. Ganbarov K. (2023). Biological activity of chalcones as carbonyl compound derivatives. Adv. Biol. Earth Sci. 8 (1), 19–26.

[B5] Chen C. X. Zhu J. Zeng Z. (2022). Use of ultrasound to observe mycosis fungoides: a case report and review of literature. Curr. Med. Imaging 18 (7), 771–775. 10.2174/1573405617666211208121419 34879810

[B6] Daina A. Michielin O. Zoete V. J. D. O. S. (2017). SwissADME: a free web tool to evaluate pharmacokinetics, drug-likeness and medicinal chemistry friendliness of small molecules. Sci. Rep. 7, 42717. 10.1038/srep42717 28256516 PMC5335600

[B7] DeLano W. L. (2002). The PyMOL molecular graphics system. Avaliable at: http://www.pymol.org (Accessed November 2, 2023).

[B8] Dhadda S. Goswami P. G. Sharma H. (2022). “Green synthesis of chalcone derivatives using chalcones as precursor,” in Green chemistry-new perspectives (IntechOpen). 10.5772/intechopen.103959

[B9] Dhankhar N. Kumar J. (2023). Impact of increasing pesticides and fertilizers on human health: a review. Mat. Today Proc. 10.1016/j.matpr.2023.03.766

[B10] Discovery Studio Visualization (2016). Biovia. Avaliable at: http://accelrys.com/products/collaborativescience/biovia-discovery-studio/visualization-download.php (Accessed November 2, 2023).

[B11] Dong X. Chen J. Jiang C. Liu T. Hu Y. (2009). Design, synthesis and biological evaluation of prenylated chalcones as vasorelaxant agents. Arch. Pharm. Chem. Life Sci. 342, 428–432. 10.1002/ardp.200800229 19544479

[B12] Escribano-Ferrer E. Queralt Regue J. Garcia-Sala X. Boix Montanes A. Lamuela-Raventos R. M. (2019). *In vivo* anti-inflammatory and antiallergic activity of pure naringenin, naringenin chalcone, and quercetin in mice. J. Nat. Prod. 82 (2), 177–182. 10.1021/acs.jnatprod.8b00366 30688453

[B13] Espinoza-Hicks J. C. Chacón-Vargas K. F. Hernández-Rivera J. L. Nogueda-Torres B. Tamariz J. Sánchez-Torres L. E. (2019). Novel prenyloxy chalcones as potential leishmanicidal and trypanocidal agents: design, synthesis and evaluation. Eur. J. Med. Chem. 167, 402–413. 10.1016/j.ejmech.2019.02.028 30784876

[B14] Farooq S. Ngaini Z. (2019). Recent synthetic methodologies for chalcone synthesis (2013-2018). Curr. Organocatalysis 6 (3), 184–192. 10.2174/2213337206666190306155140

[B15] Gaonkar S. L. Vignesh U. N. (2017). Synthesis and pharmacological properties of chalcones: a review. Res. Chem. Intermed. 43, 6043–6077. 10.1007/s11164-017-2977-5

[B16] Gedye R. Smith F. Westaway K. Ali H. Baldisera L. Laberge L. (1986). The use of microwave ovens for rapid organic synthesis. Tetrahedron Lett. 27 (3), 279–282. 10.1016/S0040-4039(00)83996-9

[B17] Giguere R. J. Bray T. L. Duncan S. M. Majetich G. (1986). Application of commercial microwave ovens to organic synthesis. Tetrahedron Lett. 27 (41), 4945–4948. 10.1016/S0040-4039(00)85103-5

[B18] Guida A. Lhouty M. H. Tichit D. Figueras F. Geneste P. (1997). Hydrotalcites as base catalysts. Kinetics of Claisen-Schmidt condensation, intramolecular condensation of acetonylacetone and synthesis of chalcone. Appl. Catal. A Gen. 164 (1-2), 251–264. 10.1016/S0926-860X(97)00175-0

[B19] Hartman C. B. Dube P. S. Legoabe L. J. Van Pelt N. Matheeussen A. Caljon G. (2024). Novel quinoline derivatives with broad‐spectrum antiprotozoal activities. Arch. Pharm., e2300319. 10.1002/ardp.202300319 38396284

[B20] Hu D. Zhang N. Zhou Q. Zhou Y. Gong C. Zhang Y. (2023). Synthesis and biological activities of novel chalcone derivatives containing pyrazole oxime ethers. Fitoterapia 166, 105458. 10.1016/j.fitote.2023.105458 36796458

[B21] Iwashina T. (2000). The structure and distribution of the flavonoids in plants. J. Plant Res. 113 (3), 287–299. 10.1007/PL00013940

[B22] Jain S. Kumar S. Lamba B. Y. Patra J. Mahindroo N. (2021). Nanocatalysts: applications in synthesis of chalcones-a review. Synth. Commun. 51 (1), 1–12. 10.1080/00397911.2020.1817941

[B23] Jiang M. Chen S. Lu X. Guo H. Chen S. Yin X. (2023). Integrating genomics and metabolomics for the targeted Discovery of new cyclopeptides with antifungal activity from a marine-derived fungus *Beauveria felina* . J. Agric. Food Chem. 71, 9782–9795. 10.1021/acs.jafc.3c02415 37310400

[B24] Jin H. S. Zhang S. Q. Sun R. Dou F. Zhao L. M. (2014). Introduction of prenyl fragment into chalcones through *α*-regioselective 1, 2-addition in THF. RSC Adv. 4 (42), 21810–21814. 10.1039/C4RA03301A

[B25] Jung J. C. Lee Y. Min D. Jung M. Oh S. (2017). Practical synthesis of chalcone derivatives and their biological activities. Molecules 22 (11), 1872. 10.3390/molecules22111872 29104222 PMC6150315

[B26] Keiko N. A. Vchislo N. V. (2016a). Synthesis of diheteroatomic five‐membered heterocyclic compounds from α, β‐unsaturated aldehydes. Asian J. Org. Chem. 5 (10), 1169–1197. 10.1002/ajoc.201600227

[B27] Keiko N. A. Vchislo N. V. (2016b). α, β‐unsaturated aldehydes in the synthesis of five‐membered heterocyclic compounds with one heteroatom: recent advances from developments in metal and organocatalysis. Asian J. Org. Chem. 5 (4), 439–461. 10.1002/ajoc.201600010

[B28] Kundu A. Mandal A. Saha S. Prabhakaran P. Walia S. (2020). Fungicidal activity and molecular modeling of fusarubin analogues from *Fusarium oxysporum* . Toxicol. Environ. Chem. 102 (1-4), 78–91. 10.1080/02772248.2020.1770253

[B29] McGovern R. J. (2015). Management of tomato diseases caused by *Fusarium oxysporum* . Crop Prot. 73, 78–92. 10.1016/j.cropro.2015.02.021

[B30] Nasir Abbas Bukhari S. Jasamai M. Jantan I. Ahmad W. (2013). Review of methods and various catalysts used for chalcone synthesis. Mini Rev. Org. Chem. 10 (1), 73–83. 10.2174/1570193X11310010006

[B31] Nene Y. L. Thapliyal P. N. (1979). “Fungicides in plant disease control,” in Evaluation of fungicides. Editors Nene Y. L. Thapliyal P. N. (New Delhi: Oxford and IBH Publishing Co.), 406–428.

[B32] Neves M. P. Lima R. T. Choosang K. Pakkong P. De São José Nascimento M. Vasconcelos M. H. (2012). Synthesis of a natural chalcone and its prenyl analogs- Evaluation of tumor cell growth-inhibitory activities, and effects on cell cycle and apoptosis. Chem. Biodivers. 9 (6), 1133–1143. 10.1002/cbdv.201100190 22700231

[B33] Nielsen S. F. Larsen M. Boesen T. Schønning K. Kromann H. (2005). Cationic chalcone antibiotics. Design, synthesis, and mechanism of action. J. Med. Chem. 48 (7), 2667–2677. 10.1021/jm049424k 15801857

[B34] Nowakowska Z. (2007). A review of anti-infective and anti-inflammatory chalcones. Eur. J. Med. Chem. 42 (2), 125–137. 10.1016/j.ejmech.2006.09.019 17112640

[B35] Osorio M. E. Quiroz K. A. Carvajal M. A. Vergara A. P. Sánchez E. Y. González C. E. (2016). Synthesis, anti-phytopathogenic and DPPH radical scavenging activities of C-prenylated acetophenones and benzaldehydes. J. Chil. Chem. Soc. 61 (3), 3095–3101. 10.4067/s0717-97072016000300018

[B36] Passalacqua T. G. Dutra L. A. De Almeida L. Velásquez A. M. A. Torres Esteves F. A. Yamasaki P. R. (2015). Synthesis and evaluation of novel prenylated chalcone derivatives as anti-leishmanial and anti-trypanosomal compounds. Bioorg. Med. Chem. Lett. 25 (16), 3342–3345. 10.1016/j.bmcl.2015.05.072 26055530

[B37] Piñero J. Temporal R. M. Silva-Gonçalves A. J. Jiménez I. A. Bazzocchi I. L. Oliva A. (2006). New administration model of trans-chalcone biodegradable polymers for the treatment of experimental leishmaniasis. Acta trop. 98 (1), 59–65. 10.1016/j.actatropica.2006.02.001 16529707

[B38] Rajendran G. Bhanu D. Aruchamy B. Ramani P. Pandurangan N. Bobba K. N. (2022). Chalcone: a promising bioactive scaffold in medicinal chemistry. Pharm. (Basel) 15 (10), 1250. 10.3390/ph15101250 PMC9607481 36297362

[B39] Rammohan A. Reddy J. S. Sravya G. Rao C. N. Zyryanov G. V. (2020). Chalcone synthesis, properties and medicinal applications: a review. Environ. Chem. Lett. 18, 433–458. 10.1007/s10311-019-00959-w

[B40] Rani M. S. Kalyani N. C. Murthy C. Bhasker N. Reddy B. V. S. (2019). Piperidine mediated synthesis of prenylated chalcones and 8-substituted-2, 5-dihydro-2-(4-tolybenzo)-5-(3-methylbut-2-enyloxy) phenol-1, 5-benzothiazepines and its derivatives as anticancer agents. Rasayan J. Chem. 12 (2), 796–802. 10.31788/RJC.2019.1224078

[B41] Reddy N. P. Aparoy P. Reddy T. C. M. Achari C. Sridhar P. R. Reddanna P. (2010). Design, synthesis, and biological evaluation of prenylated chalcones as 5-LOX inhibitors. Bioorg. Med. Chem. 18 (16), 5807–5815. 10.1016/j.bmc.2010.06.107 20667741

[B42] Remesal E. Landa B. B. Jimenez-Gasco M. D. M. Navas-Cortés J. A. (2013). Sequence variation in two protein-coding genes correlates with mycelial compatibility groupings in Sclerotium rolfsii. Phytopathology 103 (5), 479–487. 10.1094/PHYTO-07-12-0151-R 23301814

[B43] Sahoo B. M. Banik B. K. Kumar B. V. V. R. Panda K. C. Tiwari A. Tiwari V. (2023). Microwave induced green synthesis: sustainable Technology for efficient development of bioactive pyrimidine scaffolds. Curr. Med. Chem. 30 (9), 1029–1059. 10.2174/0929867329666220622150013 35733315

[B44] Sheridan R. P. Maiorov V. N. Holloway M. K. Cornell W. D. Gao Y.-D. (2010). Drug-like density: a method of quantifying the bindability of a protein target based on a very large set of pockets and drug-like ligands from the Protein Data Bank. J. Chem. Inf. Model. 50, 2029–2040. 10.1021/ci100312t 20977231

[B45] Sugamoto K. Matsusita Y. I. Matsui K. Kurogi C. Matsui T. (2011). Synthesis and antibacterial activity of chalcones bearing prenyl or geranyl groups from *Angelica keiskei* . Tetrahedron 67 (29), 5346–5359. 10.1016/j.tet.2011.04.104

[B46] Tandel S. Patel N. C. Kanvah S. Patel P. N. (2022). An efficient protocol for the synthesis of novel hetero-aryl chalcone: a versatile synthon for several heterocyclic scaffolds and sensors. J. Mol. Struct. 1269, 133808. 10.1016/j.molstruc.2022.133808

[B47] Verma S. Srivastava A. K. Pandey O. P. (2018). A review on chalcones synthesis and their biological activity. PharmaTutor 6 (2), 22–39. 10.29161/PT.v6.i2.2018.22

[B48] Vogel S. Heilmann J. (2008). Synthesis, cytotoxicity, and antioxidative activity of minor prenylated chalcones from Humulus lupulus. J. Nat. Prod. 71 (7), 1237–1241. 10.1021/np800188b 18611049

[B49] Wang S. Huang Z. Wan Q. Feng S. Xie X. Zhang R. (2020). Comparative genomic and metabolomic analyses of two *Pseudomonas aeruginosa* strains with different antifungal activities. Front. Microbiol. 11, 1841. 10.3389/fmicb.2020.01841 32849439 PMC7412747

[B50] Wang S. Li C. Zhang L. Sun B. Cui Y. Sang F. (2023). Isolation and biological activity of natural chalcones based on antibacterial mechanism classification. Bioorg. Med. Chem. 117454, 117454. 10.1016/j.bmc.2023.117454 37659218

[B51] Yanti Y. Hamid H. Yaherwandi R. (2021). Biological control of *Sclerotium rolfsii* on tomato seedlings using *Bacillus* spp. *consortium* . Earth Environ. Sci. 741 (1), 012063. 10.1088/1755-1315/741/1/012063

[B52] Yazaki K. Sasaki K. Tsurumaru Y. (2009). Prenylation of aromatic compounds, a key diversification of plant secondary metabolites. Phytochemistry 70 (15-16), 1739–1745. 10.1016/j.phytochem.2009.08.023 19819506

[B53] Yousefian M. Hashemi M. Eskandarpour V. Hadizadeh F. Zarghi A. Ghodsi R. (2024). Design, synthesis, biological evaluation and molecular docking study of novel chalcone-based hydroxamic acids possessing a central 2, 4-dimethy pyrrole linker as potential HDAC (Histone Deacetylase) inhibitors and anticancer agents. J. Mol. Struct. 1305, 137749. 10.1016/j.molstruc.2024.137749

[B54] Zhang N. Zeng W. Sun Z. Zhou Q. Meng K. Hu Y. (2024). Design, synthesis, and bioactivity studies of chalcone derivatives containing [1, 2, 4]-triazole-[4, 3-a]-pyridine. Fitoterapia 172, 105739. 10.1016/j.fitote.2023.105739 37952763

[B55] Zhang Z. Zhang W. Hou Z. W. Li P. Wang L. (2023). Electrophilic halospirocyclization of N-benzylacrylamides to access 4-halomethyl-2-azaspiro [4.5] decanes. J. Org. Chem. 88 (19), 13610–13621. 10.1021/acs.joc.3c01315 37694951

[B56] Zhou K. Yang S. Li S. M. (2021). Naturally occurring prenylated chalcones from plants: structural diversity, distribution, activities and biosynthesis. Nat. Prod. Rep. 38 (12), 2236–2260. 10.1039/d0np00083c 33972962

