# Supplementary material for: Green synthesis, structure–activity relationships, in silico molecular docking, and antifungal activities of novel prenylated chalcones
Source: Front Chem. 2024 Apr 26;12:1389848. doi: 10.3389/fchem.2024.1389848 (PMC11093228; doi:10.3389/fchem.2024.1389848)
Supplement: Supplementary file 1 [file DataSheet1.PDF]

Spectral analysis of synthesized prenylated chalcones (5A-5P)

1. 2'-Hydroxy-3-nitro-5'-*O*-prenylchalcone (5A)

Orange solid. IR (KBr, cm^{-1}): 1677 (C=O), 1562 (CH=CH); ^{1}H-NMR (400 MHz, $CDCl_3$): δ 1.73 (3H, s, prenyl-CH_3), 1.80 (3H, s, prenyl-CH_3), 4.54 (2H, d, J= 6.8 Hz, H-1"), 5.49 (1H, t, J= 6.8 Hz, H-2"), 6.97 (1H, d, J= 9.2 Hz, H-3'), 7.08 (1H, dd, J= 9.2 & J= 3.2 Hz, H-4'), 7.33 (1H, d, J= 3.2 Hz, H-6'), 7.57 (1H, t, J= 8 Hz & 8 Hz, H-5), 7.67 (1H, d, J= 16 Hz, H-α), 7.75 (1H, d, J= 16 Hz, H-β), 7.86 ((1H, d, J= 8 Hz, H-6), 8.22 (1H, m, H-4), 8.44 (1H, m, H-2), 12.18 (1H, s, Chelated OH); ^{13}C-NMR (100 MHz, $CDCl_3$): δ 18.21 (prenyl-CH_3), 25.75 (prenyl-CH_3), 65.38 (C-1"), 115.09 (C-3' & C-4'), 119.11 (C-2"), 119.44 (C-1'), 121.52 (C-6), 122.21 (C-4), 124.14 (C-2), 129.85 (C-α, C-5 & C-6'), 134.08 (C-1), 137.29 (C-3), 138.68 (C-3"), 148.66 (C-β), 152.65 (C-5'), 152.98 (C-2'), 190.82 (C=O). HR-MS for $C_{20}H_{19}NO_5$ $[M+H]^+$ *m/z*: Calcd 354.1336; observed 354.1733.

2. 2'-Hydroxy-2,6-dichloro-5'-*O*-prenylchalcone (5B)

Bright orange solid. IR (KBr, cm^{-1}): 1647 (C=O), 1549 (CH=CH); ^{1}H-NMR (400 MHz, $CDCl_3$): δ 1.73 (3H, s, prenyl-CH_3), 1.79 (3H, s, prenyl-CH_3), 4.49 (2H, d, J= 6.8 Hz, H-1"), 5.47 (1H, t, J= 6.8 Hz, H-2"), 6.97 (1H, d, J= 8.8 Hz, H-3'), 7.17 (1H, dd, J= 8.8 & J= 2.8 Hz, H-4'), 7.23(1H, m, H-4), 7.31 (1H, d, J= 2.8 Hz, H-6'), 7.40 (2H, d, J= 8 Hz, H-5 & H-3), 7.75 (1H, d, J=16 Hz, H-α), 7.95 (1H, d, J= 16 Hz, H-β), 12.20 (1H, s, Chelated OH); ^{13}C-NMR (100 MHz, $CDCl_3$): δ 18.19 (prenyl-CH_3), 25.78 (prenyl-CH_3), 65.65 (C-1"), 101.66 (C-3'), 108.12 (C-4'), 113.95 (C-1'), 117.77 (C-α), 118.54 (C-2"), 128.93 (C-1), 129.89 (C-3 and C-5), 131.49 (C-6'), 132.41 (C-4), 135.22 (C-2 and C-6), 139.15 (C-3"), 144.12 (C-β), 150.90, (C-5'), 157.95 (C-2'), 193.29 (C=O). HR-MS for $C_{20}H_{18}Cl_2O_3$ $[M+H]^+$ *m/z*: Calcd 377.0705; observed 377.1002.

3. 2'-Hydroxy-2,4-dichloro-5'-*O*-prenylchalcone (5C)

Orange solid. IR (KBr, cm^{-1}): 1677 (C=O), 1564 (CH=CH); ^{1}H-NMR (400 MHz, $CDCl_3$): δ 1.75 (3H, s, prenyl-CH_3), 1.81 (3H, s, prenyl-CH_3), 4.51 (2H, d, J= 6.8 Hz, H-1"), 5.49 (1H, t, J= 6.8 Hz, H-2"), 6.98 (1H, d, J= 8.8 Hz, H-3'), 7.18 (1H, dd, J= 8.8 & J= 3.2 Hz, H-4'), 7.32 (1H, dd, J= 8.8 & J= 2.8 Hz, H-5), 7.35 (1H, d, J= 2.8 Hz, H-3), 7.49 (1H, d, J= 3.2 Hz, H-6'), 7.54 (1H, d, J=15.6 Hz, H-α), 7.68 (1H, d, J= 8.8 Hz, H-6), 8.20 (1H, d, J= 16 Hz, H-β), 12.21 (1H, s, Chelated OH); ^{13}C-NMR (100 MHz, $CDCl_3$): δ 18.21 (prenyl-CH_3), 25.80 (prenyl-CH_3), 65.72(C-1"), 114.27 (C-3'), 119.26 (C-4'), 119.38 (C-1'), 123.06 (C-α) 124.78 (C-2"), 127.57 (C-1), 128.50 (C-6), 130.18 (C-5), 131.45 (C-6'), 136.22 (C-2), 136.82 (C-3), 138.68 (C-4), 139.77 (C-3"), 144.12 (C-β), 150.89, (C-5'), 157.89 (C-2'), 192.82 (C=O). HR-MS for $C_{20}H_{18}Cl_2O_3$ $[M+H]^+$ *m/z*: Calcd 377.0705; observed 377.0768.

4. 2'-Hydroxy-3-chloro-5'-*O*-prenylchalcone (5D)

Red solid. IR (KBr, cm^{-1}): 1677 (C=O), 1546 (CH=CH); ^{1}H-NMR (400 MHz, $CDCl_3$): δ 1.77 (3H, s, prenyl-CH_3), 1.82 (3H, s, prenyl-CH_3), 4.53 (2H, d, J= 6.8 Hz, H-1"), 5.50 (1H, t, J= 6.8 Hz, H-2"), 6.97 (1H, d, J= 9.2 Hz, H-3'), 7.17 (1H, dd, J= 9.2 & J= 3.2 Hz, H-4'), 7.40 (3H, m, H-6, H-5 & H-4), 7.51 (1H, m, H-2), 7.57 (1H, d, J=15.6 Hz, H-α), 7.64 (1H, m, H-6'), 7.82 (1H,

d, J= 16 Hz, H-β), 12.26 (1H, s, Chelated OH); ^{13}C-NMR (100 MHz, $CDCl_3$): δ 18.21 (prenyl-CH_3), 25.79 (prenyl-CH_3), 65.22 (C-1''), 117.61 (C-3'), 119.45 (C-4'), 122.38 (C-1'), 125.88 (C-α), 126.82 (C-2''), 127.39 (C-1), 128.28 (C-6), 128.85 (C-6'), 132.95 (C-5), 133.23 (C-2), 134.64 (C-4), 136.37 (C-3''), 138.42 (C-β), 141.78 (C-3), 150.81, (C-5'), 156.19 (C-2'), 191.25 (C=O). HR-MS for $C_{20}H_{19}ClO_3$ $[M+H]^+$ *m/z*: Calcd 343.1095; observed 343.0899.

5. 2'-Hydroxy-4-bromo-5'-*O*-prenylchalcone (5E)

Bright red solid. IR (KBr, cm^{-1}): 1677 (C=O), 1531 (CH=CH); ^{1}H-NMR (400 MHz, $CDCl_3$): δ 1.76 (3H, s, prenyl-CH_3), 1.81 (3H, s, prenyl-CH_3), 4.51 (2H, d, J= 6.8 Hz, H-1''), 5.49 (1H, t, J= 6.8 Hz, H-2''), 6.96 (1H, d, J= 9.2 Hz, H-3'), 7.16 (1H, dd, J= 9.2 & J= 2.8 Hz, H-4'), 7.37 (1H, d, J= 2.8 Hz, H-6'), 7.51 (2H, m, H-6 & H-2), 7.55 (2H, m, H-5 & H-3), 7.56 (1H, d, J=15.2 Hz, H-α), 7.83 (1H, d, J= 16 Hz, H-β), 12.26 (1H, s, Chelated OH); ^{13}C-NMR (100 MHz, $CDCl_3$): δ 18.24 (prenyl-CH_3), 25.85 (prenyl-CH_3), 65.75 (C-1''), 114.37 (C-1'), 119.22 (C-3'), 119.40 (C-2 & C-6), 119.57 (C-2''), 120.68 (C-4'), 124.56 (C-α), 125.25 (C-6'), 129.92 (C-1), 132.28 (C-3 & C-5), 133.45 (C-4), 138.76 (C-3''), 144.01 (C-β), 150.89 (C-5'), 157.85 (C-2'), 193.08 (C=O). HR-MS for $C_{20}H_{19}BrO_3$ $[M+H]^+$ *m/z*: Calcd 387.0590; observed 387.0599.

6. 2'-Hydroxy-3-bromo-5'-*O*-prenylchalcone (5F)

Yellow solid. IR (KBr, cm^{-1}): 1676 (C=O), 1549 (CH=CH); ^{1}H-NMR (400 MHz, $CDCl_3$): δ 1.78 (3H, s, prenyl-CH_3), 1.82 (3H, s, prenyl-CH_3), 4.53 (2H, d, J= 6.8 Hz, H-1''), 5.50 (1H, t, J= 6.8 Hz, H-2''), 6.97 (1H, d, J= 9.2 Hz, H-3'), 7.17 (1H, dd, J= 9.2 & J= 2.8 Hz, H-4'), 7.32 (1H, t, J= 8 Hz & J= 7.6, H-5), 7.37 (1H, d, J= 2.8 Hz, H-6'), 7.55 (2H, m, H-6 & H-4), 7.56 (1H, d, J=16 Hz, H-α), 7.80 (1H, m, H-2), 7.81 (1H, d, J= 16 Hz, H-β), 12.26 (1H, s, Chelated OH); ^{13}C-NMR (100 MHz, $CDCl_3$): δ 18.26 (prenyl-CH_3), 25.85 (prenyl-CH_3), 65.80 (C-1''), 114.31 (C-1'), 119.47 (C-3' & C-4'), 121.45 (C-2''), 123.15 (C-6), 124.47 (C-4), 124.76 (C-2), 130.70 (C-α, C-5 & C-6'), 133.58 (C-1), 136.64 (C-3), 138.69 (C-3''), 143.58 (C-β), 150.93 (C-5'), 157.89 (C-2'), 192.97 (C=O). HR-MS for $C_{20}H_{19}BrO_3$ $[M+H]^+$ *m/z*: Calcd 387.0590; observed 387.0891.

7. 2'-Hydroxy-2-bromo-5'-*O*-prenylchalcone (5G)

Yellow solid. IR (KBr, cm^{-1}): 1677 (C=O), 1546 (CH=CH); ^{1}H-NMR (400 MHz, $CDCl_3$): δ 1.73 (3H, s, prenyl-CH_3), 1.79 (3H, s, prenyl-CH_3), 4.52 (2H, d, J= 6.8 Hz, H-1''), 5.47 (1H, t, J= 6.8 Hz, H-2''), 6.94 (1H, d, J= 9.2 Hz, H-3'), 7.05 (1H, dd, J= 9.2 & J= 2.8 Hz, H-4'), 7.21 (1H, ddd, J= 8 Hz, J= 7.6 Hz, J= 1.6 Hz, H-5), 7.31 (2H, m, H-6' & H-4), 7.52 (1H, d, J=16 Hz, H-α), 7.61 (1H, dd, J= 8 Hz & J= 1.2 Hz, H-6), 7.68 (1H, dd, J= 8 Hz & J= 1.6 Hz, H-3), 8.00 (1H, d, J= 16 Hz, H-β), 12.30 (1H, s, Chelated OH); ^{13}C-NMR (100 MHz, $CDCl_3$): δ 18.18 (prenyl-CH_3), 25.70 (prenyl-CH_3), 65.90 (C-1''), 115.02 (C-1'), 115.19 (C-3'), 119.36 (C-2''), 119.41 (C-4'), 120.99 (C-3 and C-4), 125.82 (C-α), 127.56 (C-1), 129.47 (C-2), 130.85 (C-5 and C-6'), 135.33 (C-6), 138.44 (C-3''), 140.53 (C-β), 152.35 (C-5'), 152.88 (C-2'), 191.51 (C=O). HR-MS for $C_{20}H_{19}BrO_3$ $[M+H]^+$ *m/z*: Calcd 387.0590; observed 387.0648.

8. 2'-Hydroxy-4-nitro-5'-*O*-prenylchalcone (5H)

Red solid. IR (KBr, cm^{-1}): 1677 (C=O), 1565 (CH=CH); ^{1}H-NMR (400 MHz, $CDCl_3$): δ 1.76 (3H, s, prenyl-CH_3), 1.82 (3H, s, prenyl-CH_3), 4.51 (2H, d, J= 6.8 Hz, H-1"), 5.50 (1H, t, J= 6.8 Hz, H-2"), 6.96 (1H, d, J= 9.2 Hz, H-3'), 7.16 (1H, dd, J= 9.2 & J= 2.8 Hz, H-4'), 7.38 (1H, d, J= 2.8 Hz, H-6'), 7.52 (2H, m, H-6 & H-2), 7.55 (2H, m, H-5 & H-3), 7.57 (1H, d, J=15.2 Hz, H-α), 7.83 (1H, d, J= 16 Hz, H-β), 12.26 (1H, s, Chelated OH); ^{13}C-NMR (100 MHz, $CDCl_3$): δ 18.24 (prenyl-CH_3), 25.85 (prenyl-CH_3), 65.75 (C-1"), 114.37 (C-1'), 119.22 (C-3'), 119.41 (C-2 & C-6), 119.57 (C-2"), 120.68 (C-4'), 124.56 (C-α), 125.25 (C-6'), 129.92 (C-1), 132.28 (C-3 & C-5), 133.45 (C-4), 138.76 (C-3"), 144.01 (C-β), 150.89 (C-5'), 157.85 (C-2'), 193.08 (C=O). HR-MS for $C_{20}H_{19}NO_5$ $[M+H]^+$ *m/z*: Calcd 354.1336; observed 354.1733.

9. 2'-Hydroxy-3,4,5-trimethoxy-5'-*O*-prenylchalcone (5I)

Yellow solid. IR (KBr, cm^{-1}): 1677 (C=O), 1562 (CH=CH); ^{1}H-NMR (400 MHz, $CDCl_3$): δ 1.76 (3H, s, prenyl-CH_3), 1.81 (3H, s, prenyl-CH_3), 3.87 (9H, s, 3 × OMe) 4.57 (2H, d, J= 6.8 Hz, H-1"), 5.49 (1H, t, J= 6.8 Hz, H-2"), 6.50 (2H, m, H-4'& H-3'), 6.872 (2H, m, H-6 &H-2), 7.45 (1H, d, J= 15.2 Hz, H-α), 7.80 (1H, d, J= 15.2 Hz, H-β), 7.83 (1H, d, J= 8.4 Hz, H-6'), 13.39 (1H, s, Chelated OH); ^{13}C-NMR (100 MHz, $CDCl_3$): δ 18.23 (prenyl-CH_3), 25.81 (prenyl-CH_3), 56.22 (2 × OMe), 61.00 (OMe), 65.18 (C-1"), 101.64 (C-3'), 105.73 (C-2 and C-6), 108.31 (C-4'), 113.91 (C-1'), 118.58 (C-2"), 119.47 (C-α), 130.27 (C-1), 131.11 (C-6'), 139.25 (C-3"), 140.51 (C-4), 144.45 (C-β), 153.47 (C-3 and C-5), 165.53 (C-5'), 166.63 (C-2'), 191.54 (C=O). HR-MS for $C_{23}H_{26}O_6$ $[M+H]^+$ *m/z*: Calcd 399.1802; observed 399.1882.

10. 2'-Hydroxy-2-nitro-5'-*O*-prenylchalcone (5J)

Yellow solid. IR (KBr, cm^{-1}): 1677 (C=O), 1562 (CH=CH); ^{1}H-NMR (400 MHz, $CDCl_3$): δ 1.73 (3H, s, prenyl-CH_3), 1.82 (3H, s, prenyl-CH_3), 4.55 (2H, d, J= 6.8 Hz, H-1"), 5.45 (1H, t, J= 6.8 Hz, H-2"), 6.92 (1H, d, J= 9.2 Hz, H-3'), 7.04 (1H, dd, J= 9.2 & J= 3.2 Hz, H-4'), 7.25 (1H, ddd, J= 8 Hz, J= 7.2 Hz, J= 1.6 Hz, H-5), 7.31 (2H, m, H-4 & H-6'), 7.49 (1H, d, J=15.6 Hz, H-α), 7.63 (1H, dd, J= 8 Hz & J= 2 Hz, H-6), 7.72 (1H, dd, J= 8 Hz & J= 1.6 Hz, H-3), 8.20 (1H, d, J= 15.6 Hz, H-β), 13.34 (1H, s, Chelated OH); ^{13}C-NMR (100 MHz, $CDCl_3$): δ 18.18 (prenyl-CH_3), 25.70 (prenyl-CH_3), 65.90 (C-1"), 115.02 (C-1'), 115.20 (C-3'), 119.36 (C-2"), 119.42 (C-4'), 120.98 (C-3 and C-4), 125.82 (C-α), 127.56 (C-1), 129.47 (C-2), 130.85 (C-5 and C-6'), 135.34 (C-6), 138.44 (C-3"), 140.54 (C-β), 152.35 (C-5'), 152.88 (C-2'), 191.51 (C=O). HR-MS for $C_{20}H_{19}NO_5$ $[M+H]^+$ *m/z*: Calcd 354.1336; observed 354.1733.

11. 2'-Hydroxy-4-hydroxy-5'-*O*-prenylchalcone (5K)

Yellow solid. IR (KBr, cm^{-1}): 1677 (C=O), 1562 (CH=CH); ^{1}H-NMR (400 MHz, $CDCl_3$): δ 1.75 (3H, s, prenyl-CH_3), 1.82 (3H, s, prenyl-CH_3), 4.52 (2H, d, J= 6.8 Hz, H-1"), 5.50 (1H, t, J= 6.8 Hz, H-2"), 6.96 (1H, d, J= 9.2 Hz, H-3'), 7.16 (1H, dd, J= 9.2 & J= 2.8 Hz, H-4'), 7.38 (1H, d, J= 2.8 Hz, H-6'), 7.52 (2H, m, H-6 & H-2), 7.56 (2H, m, H-5 & H-3), 7.57 (1H, d, J=15.2 Hz, H-α), 7.84 (1H, d, J= 16 Hz, H-β), 12.26 (1H, s, Chelated OH); ^{13}C-NMR (100 MHz, $CDCl_3$): δ 18.24 (prenyl-CH_3), 25.85 (prenyl-CH_3), 65.75 (C-1"), 114.37 (C-1'), 119.22 (C-3'), 119.40 (C-2 & C-6), 119.57 (C-2"), 120.68 (C-4'), 124.56 (C-α), 125.25 (C-6'), 129.92 (C-1), 132.28 (C-3 & C-5), 133.45 (C-4), 138.76 (C-3"), 144.01 (C-β), 150.89 (C-5'), 157.85 (C-2'), 193.08 (C=O). HR-MS for $C_{20}H_{20}O_4$ $[M+H]^+$ *m/z*: Calcd 325.1434; observed 325.1256.

12. 2'-Hydroxy-3-hydroxy-5'-*O*-prenylchalcone (5L)

Yellow solid. IR (KBr, cm^{-1}): 1677 (C=O), 1567 (CH=CH); ^{1}H-NMR (400 MHz, $CDCl_3$): δ 1.73 (3H, s, prenyl-CH_3), 1.78 (3H, s, prenyl-CH_3), 4.52 (2H, d, J= 6.8 Hz, H-1"), 5.47 (1H, t, J= 6.8 Hz, H-2"), 6.87 (1H, m, H-6), 6.94 (1H, d, J= 9.2 Hz, H-3'), 7.03 (1H, dd, J= 9.2 & J= 3.2 Hz, H-4'), 7.11 (1H, d, J= 16 Hz, H-α), 7.14 (1H, d, J= 3.2 Hz, H-6'), 7.24 (2H, m, H-4 & H-2), 7.55 (1H, d, J= 16 Hz, H-β) 7.61 (1H, d, J= 3.2 Hz, H-5), 12.62 (1H, s, Chelated OH); ^{13}C-NMR (100 MHz, $CDCl_3$): δ 18.14 (prenyl-CH_3), 25.70 (prenyl-CH_3), 65.47 (C-1"), 114.32 (C-1'), 115.34 (C-3' & C-4'), 117.57 (C-2"), 119.37 (C-6), 120.41 (C-4), 120.95 (C-2), 127.16 (C-1), 129.61 (C-α, C-5 & C-6'), 136.57 (C-3), 138.48 (C-3"), 143.19 (C-β), 152.60 (C-5'), 156.49 (C-2'), 192.71 (C=O). HR-MS for $C_{20}H_{20}O_4$ $[M+H]^+$ *m/z*: Calcd 325.1434; observed 325.1261.

13. 2'-Hydroxy-4-ethoxy-5'-*O*-prenylchalcone (5M)

Yellow solid. IR (KBr, cm^{-1}): 1646 (C=O), 1562 (CH=CH); ^{1}H-NMR (400 MHz, $CDCl_3$): δ 1.44 (3H, d, OEt-CH_3), δ 1.75 (3H, s, prenyl-CH_3), 1.80 (3H, s, prenyl-CH_3), 4.08 (2H, t, OEt-CH_2), 4.56 (2H, d, J= 6.8 Hz, H-1"), 5.48 (1H, t, J= 6.8 Hz, H-2"), 6.48 (2H, m, H-4'& H-3'), 6.92 (2H, m, H-5 & H-3), 7.45 (1H, d, J=15.6 Hz, H-α), 7.59 (2H, m, H-6 & H-2), 7.81 (1H, d, J= 8.4 Hz, H-6'), 7.85 (1H, d, J= 15.6 Hz, H-β), 13.57 (1H, s, Chelated OH); ^{13}C NMR (100 MHz, $CDCl_3$): δ 14.70 (OEt-CH_3), 18.22 (prenyl-CH_3), 25.80 (prenyl-CH_3), 63.65 (OEt-CH_2), 65.13 (C-1"), 101.66 (C-3'), 108.14 (C-1'), 114.02 (C-4'), 114.89 (C-3 and C-5), 117.67 (C-α), 118.68 (C-2"), 127.33 (C-1), 130.34 (C-2 and C-6), 131.04 (C-6'), 139.13 (C-3"), 144.23 (C-β), 161.18 (C-4), 165.33 (C-5'), 166.53 (C-2'), 191.80 (C=O). HR-MS for $C_{22}H_{24}O_4$ $[M+H]^+$ *m/z*: Calcd 353.1747; observed 353.2039.

14. 2'-Hydroxy-4-methoxy-5'-*O*-prenylchalcone (5N)

Yellow solid. IR (KBr, cm^{-1}): 1646 (C=O), 1562 (CH=CH); ^{1}H-NMR (400 MHz, $CDCl_3$): δ 1.77 (3H, s, prenyl-CH_3), 1.81 (3H, s, prenyl-CH_3), 3.86 (3H, s, OMe), 4.51 (2H, d, J= 6.8 Hz, H-1"), 5.50 (1H, t, J= 6.8 Hz, H-2"), 6.95 (3H, m, H-3', H-6 & H-2), 7.14 (1H, dd, J= 9.2 & J= 3.2 Hz, H-4'), 7.40 (1H, d, J= 3.2 Hz, H-6'), 7.46 (1H, d, J= 15.6 Hz, H-α), 7.61 (2H, m, H-3 & H-5), 7.88 (1H, d, J= 15.2 Hz, H-β), 12.62 (1H, s, Chelated OH); ^{13}C-NMR (100 MHz, $CDCl_3$): δ 18.15 (prenyl-CH_3), 25.70 (prenyl-CH_3), 55.25 (C-OMe), 65.45 (C-1"), 108.17 (C-1'), 114.23 (C-3'), 117.48 (C-3 & C-5), 118.98 (C-2"), 119.26 (C-4'), 124.09 (C-α), 125.82 (C-6'), 127.39 (C-1), 130.43 (C-2 & C-6), 138.39 (C-3"), 145.27 (C-β), 150.73 (C-5'), 157.70 (C-2'), 161.91 (C-4), 193.17 (C=O). HR-MS for $C_{21}H_{22}O_4$ $[M+H]^+$ *m/z*: Calcd 339.1590; observed 339.1392.

15. 2'-Hydroxy-4-dimethylamino-5'-*O*-prenylchalcone (5O)

Brick red solid. IR (KBr, cm^{-1}): 1646 (C=O), 1562 (CH=CH); ^{1}H-NMR (400 MHz, $CDCl_3$): δ 1.77 (3H, s, prenyl-CH_3), 1.81 (3H, s, prenyl-CH_3), 3.05 (6H, s, N-CH_3×2), 4.52 (2H, d, J= 6.8 Hz, H-1"), 5.51 (1H, t, J= 6.8 Hz, H-2"), 6.69 (2H, m, H-2 & H-6), 6.94 (1H, d, J= 9.2 Hz, H-3'), 7.11 (1H, dd, J= 9.2 & J= 3.2 Hz, H-4'), 7.38 (1H, d, J= 15.2 Hz, H-α), 7.41 (1H, d, J= 3.2 Hz, H-6'), 7.55 (2H, m, H-3 & H-5), 7.90 (1H, d, J= 15.2 Hz, H-β), 12.72 (1H, s, Chelated OH); ^{13}C-NMR (100 MHz, $CDCl_3$): δ 18.22 (prenyl-CH_3), 25.82 (prenyl-CH_3), 40.05 (N-CH_3×2), 65.72

(C-1"), 108.17 (C-1'), 111.75 (C-3'), 114.26 (C-3 & C-5), 118.88 (C-2"), 119.60 (C-4'), 120.07 (C-α), 122.26 (C-6'), 123.56 (C-1), 130.78 (C-2 & C-6), 138.48 (C-3"), 146.54 (C-β), 150.07 (C-4), 152.24 (C-5'), 157.66 (C-2') 193.06 (C=O). HR-MS for $C_{22}H_{25}NO_3$ $[M+H]^+$ *m/z*: Calcd 352.1907; observed 352.2721.

16. 2'-Hydroxy-4-benzyloxy-5'-*O*-prenylchalcone (5P)

Orange solid. IR (KBr, cm^{-1}): 1646 (C=O), 1562 (CH=CH); ^{1}H-NMR (400 MHz, $CDCl_3$): δ 1.76 (3H, s, prenyl-CH_3), 1.81 (3H, s, prenyl-CH_3), 4.51 (2H, d, J= 6.8 Hz, H-1"), 5.12 (2H, s, benzyl-CH_2), 5.50 (1H, t, J= 6.8 Hz, H-2"), 6.95 (1H, d, J= 9.2 Hz, H-3'), 7.02 (2H, m, H-2 & H-6), 7.14 (1H, dd, J= 9.2 & J= 3.2 Hz, H-4'), 7.38 (6H, m, H-2''', 3''', 4''', 5'', 6''' & 6'), 7.46 (1H, d, J= 15.2 Hz, H-α), 7.61 (2H, m, H-3 & H-5), 7.88 (1H, d, J= 15.2 Hz, H-β), 12.47 (1H, s, Chelated OH); ^{13}C-NMR (100 MHz, $CDCl_3$): δ 18.22 (prenyl-CH_3), 25.79 (prenyl-CH_3), 65.21 (C-1"), 70.11 (benzyl-CH_2), 101.67 (C-3'), 108.52 (C-4'), 113.90 (C-1'), 115.35 (C-6''' and C-2'''), 117.93 (C-α), 118.65 (C-2"), 127.46 (C-1), 127.78 (C-4'''), 125.82 (C-6'), 128.19 (C-6 and C-2), 128.62 (C-5 and C-3), 130.32 (C-5'''), 131.05 (C-3'''), 136.30 (C-4), 139.16 (C-3"), 145.30 (C-β), 160.89 (C-1'''), 165.39 (C-5'), 166.54 (C-2'), 193.27 (C=O). HR-MS for $C_{27}H_{26}O_4$ $[M+H]^+$ *m/z*: Calcd 415.1903; observed 415.1981.
