# Supplementary material for: Green synthesis, structure–activity relationships, in silico molecular docking, and antifungal activities of novel prenylated chalcones
Source: Front Chem. 2024 Apr 26;12:1389848. doi: 10.3389/fchem.2024.1389848 (PMC11093228; doi:10.3389/fchem.2024.1389848)
Supplement: Supplementary file 2 [file DataSheet2.docx]

**Green synthesis, structure activity relationship, *in silico* molecular docking and antifungal activity of novel prenylated chalcones**

Rajni Godara^a^, Parshant Kaushik^a^, Kailashpati Tripathi^a,e^, Rakesh Kumar^a,f^, Virendra Singh Rana^a^, Rajesh Kumar^a^, Abhishek Mandal^a,g^, V. Shanmugam^b^, Pankaj^c^ and Najam Akhtar Shakil^a,^*

*^a^Division of Agricultural Chemicals, ^b^Division of Plant Pathology, ^c^Division of Nematology, ICAR-Indian Agricultural Research Institute, New Delhi 110012, India*

*^e^ICAR-National Research Centre on Seed Spices, Ajmer, Rajasthan 305206, India*

*^f^ICAR-Central Inland Fisheries Research Institute, Guwahati, Assam 781006, India*

*^g^ICAR-Indian Institute of Horticultural Research, Bengaluru, Karnataka 560089, India*

*Correspondence: iamshakil@gmail.com

**Supporting Information**

**Figure 1:** ^1^H NMR spectrum of compound 5A

**Figure 2:** ^13^C NMR spectrum of compound 5A

**Figure 3:** HMBC spectrum of compound 5A

**Figure 4:** HSQC spectrum of compound 5A

**Figure 5:** COSY spectrum of compound 5A

**Figure 6:** DEPT 45 spectrum of compound 5A

**Figure 7:** DEPT 90 spectrum of compound 5A

**Figure 8:** DEPT 135 spectrum of compound 5A


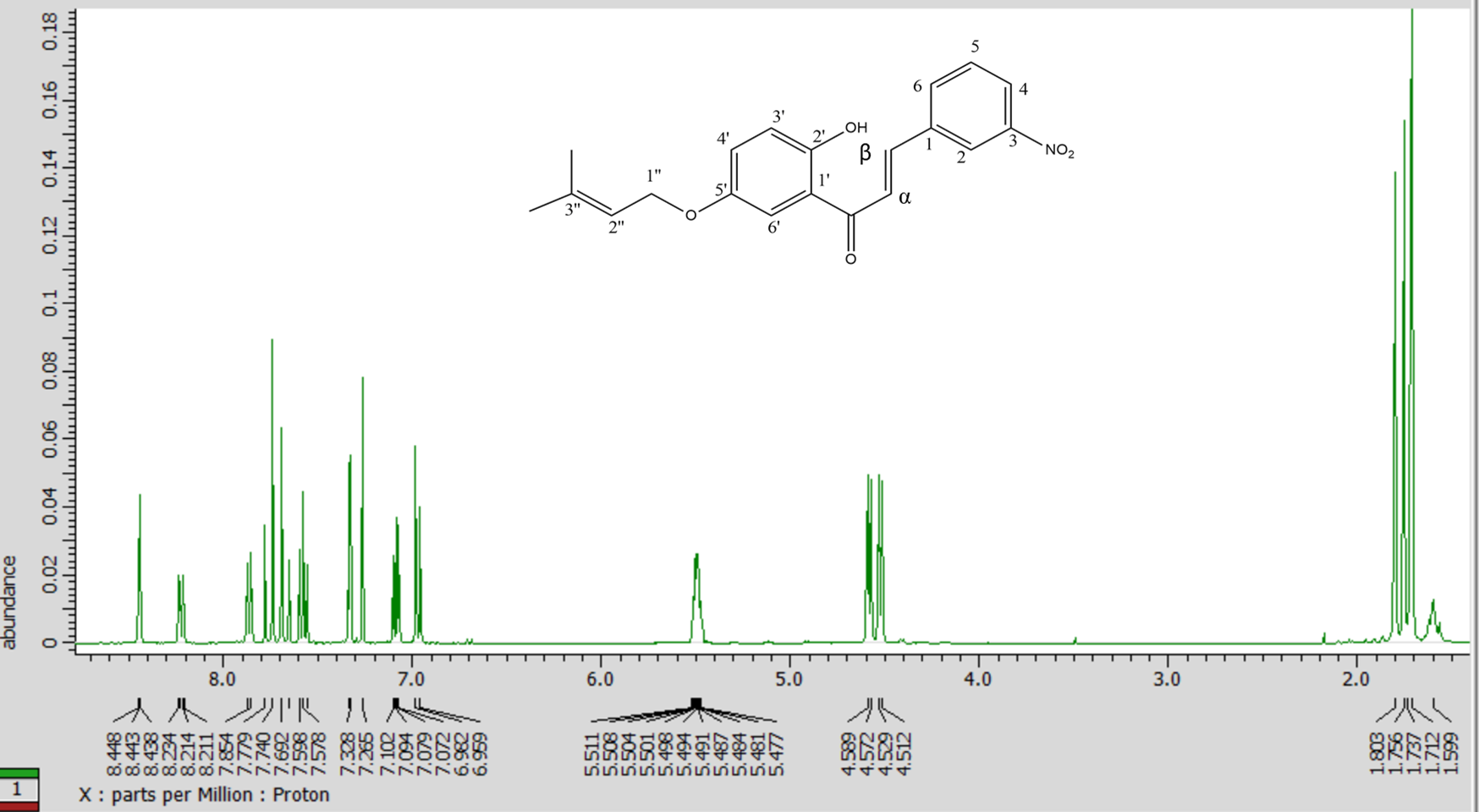


**Figure 1:** 1H-NMR (400 MHz, CDCl_3_) of compound 5A


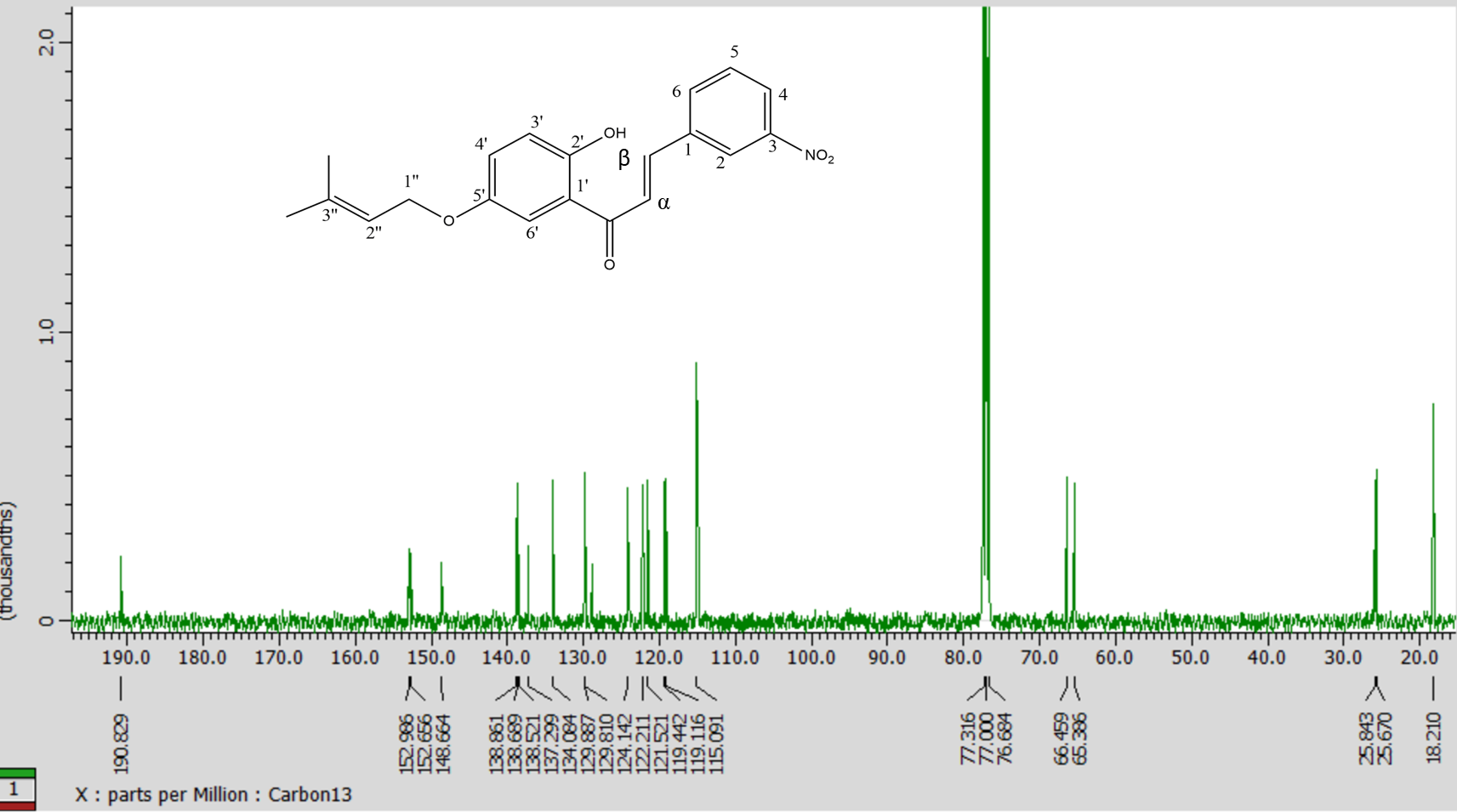


**Figure 2**: ^13^C-NMR (100.6 MHz, CDCl_3_) of compound 5A

**
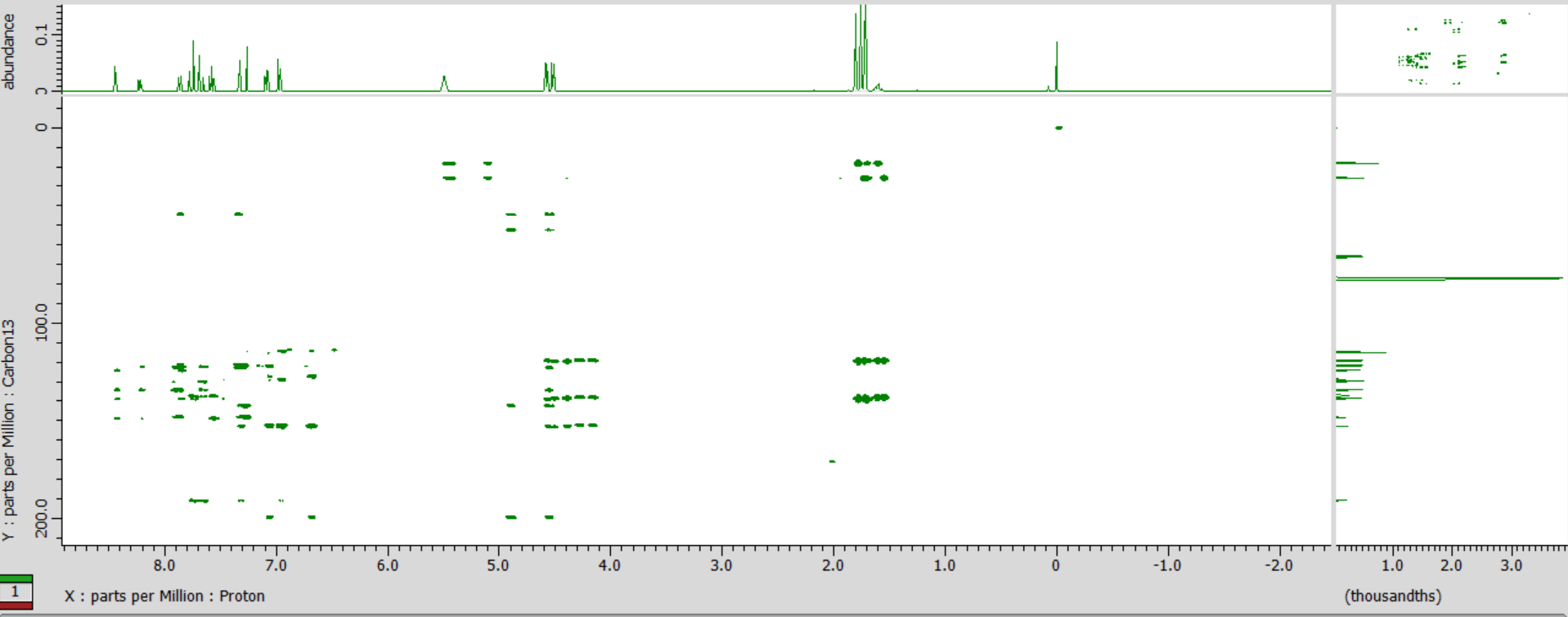
**

**Figure 3**: HMBC spectrum of compound 5A


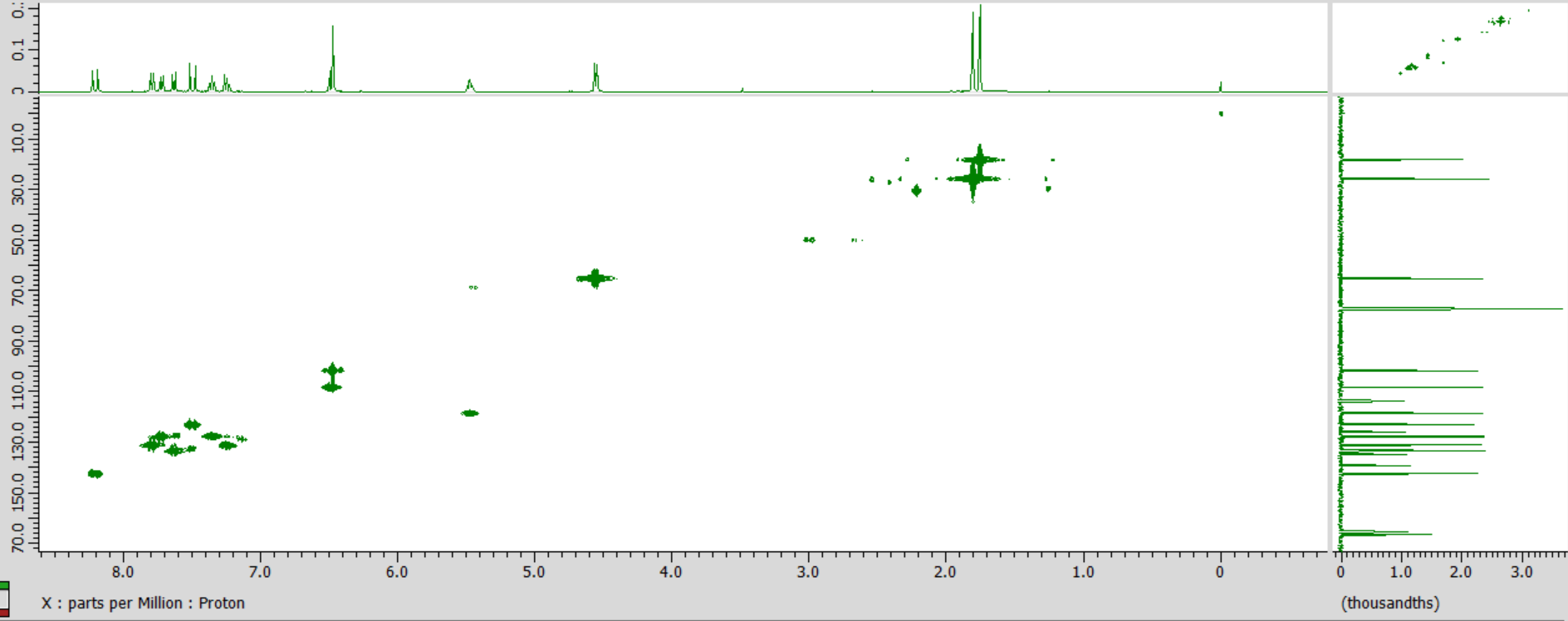


**Figure 4**: HSQC spectrum of compound 5A


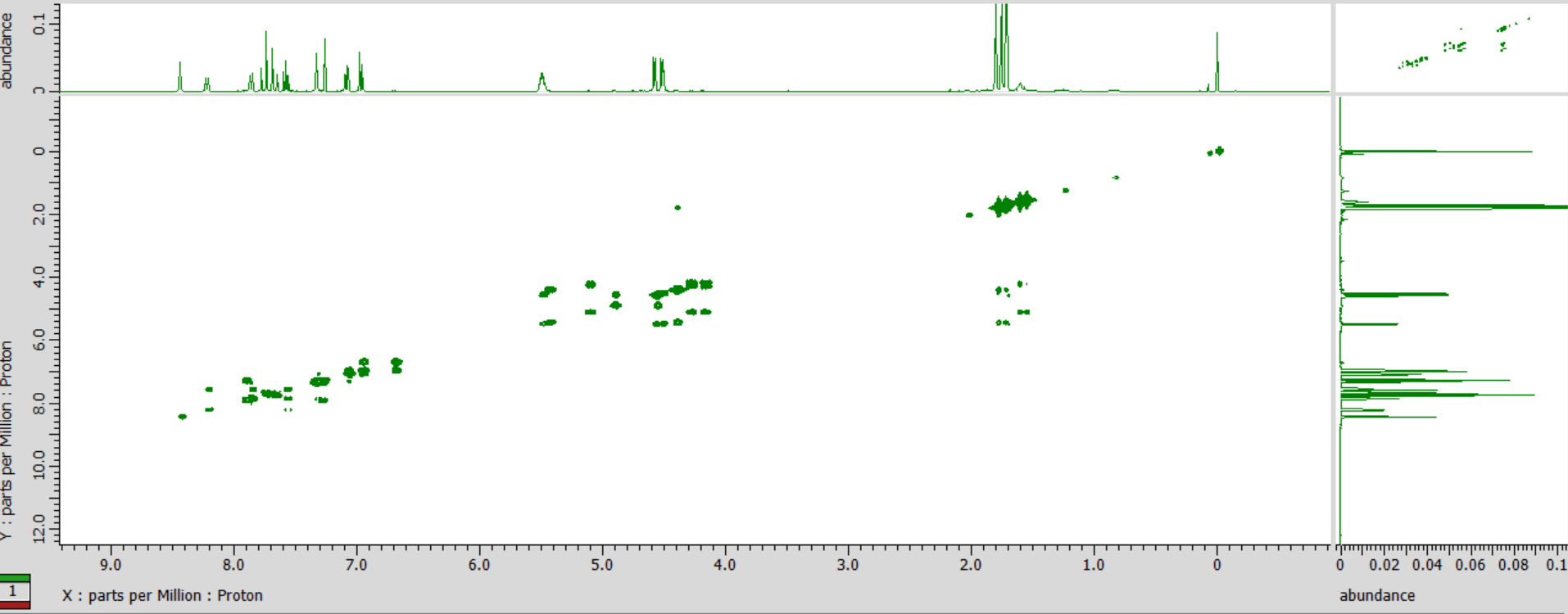


**Figure 5**: COSY spectrum of compound 5A

**
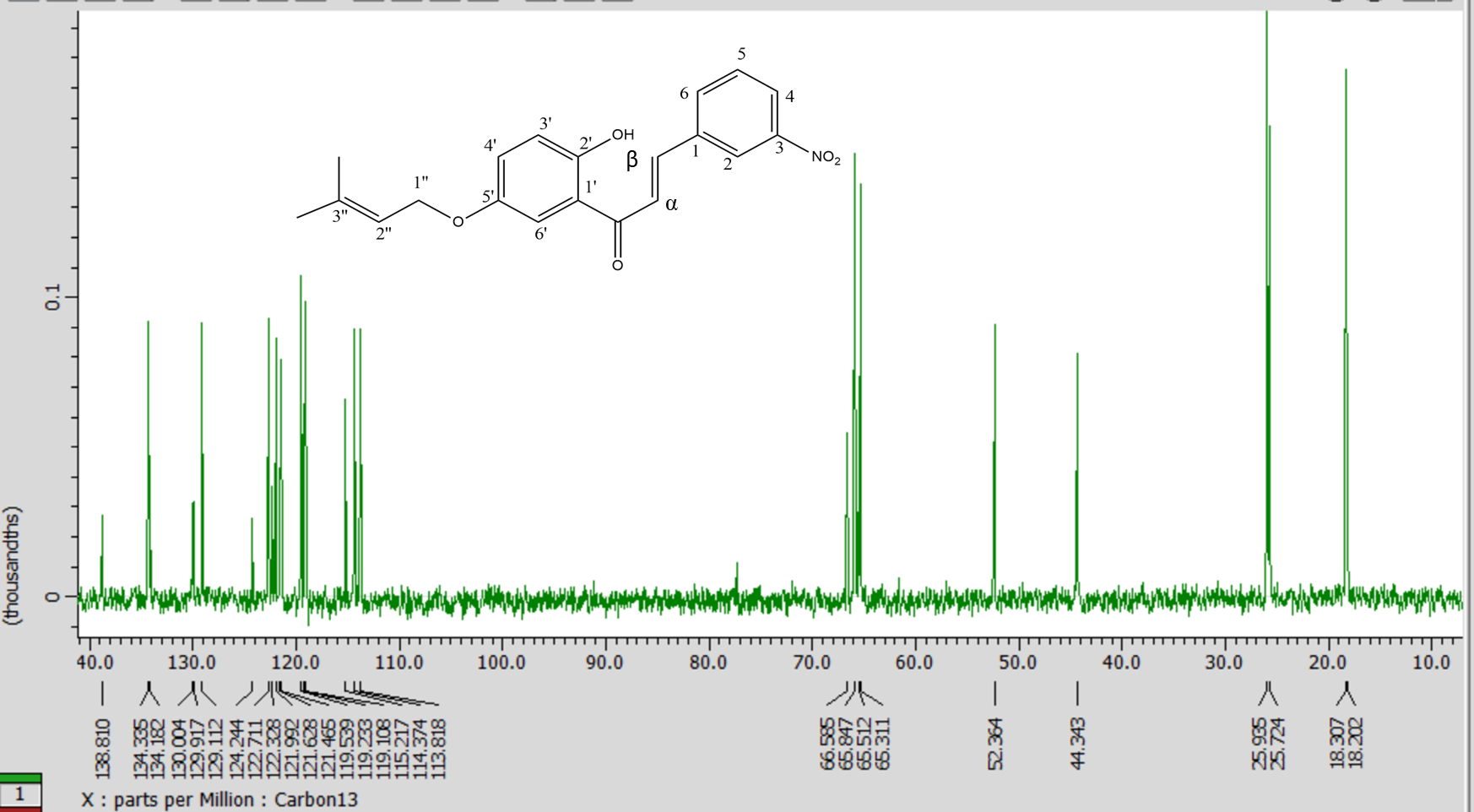
**

**Figure 6**: DEPT 45 spectrum of compound 5A


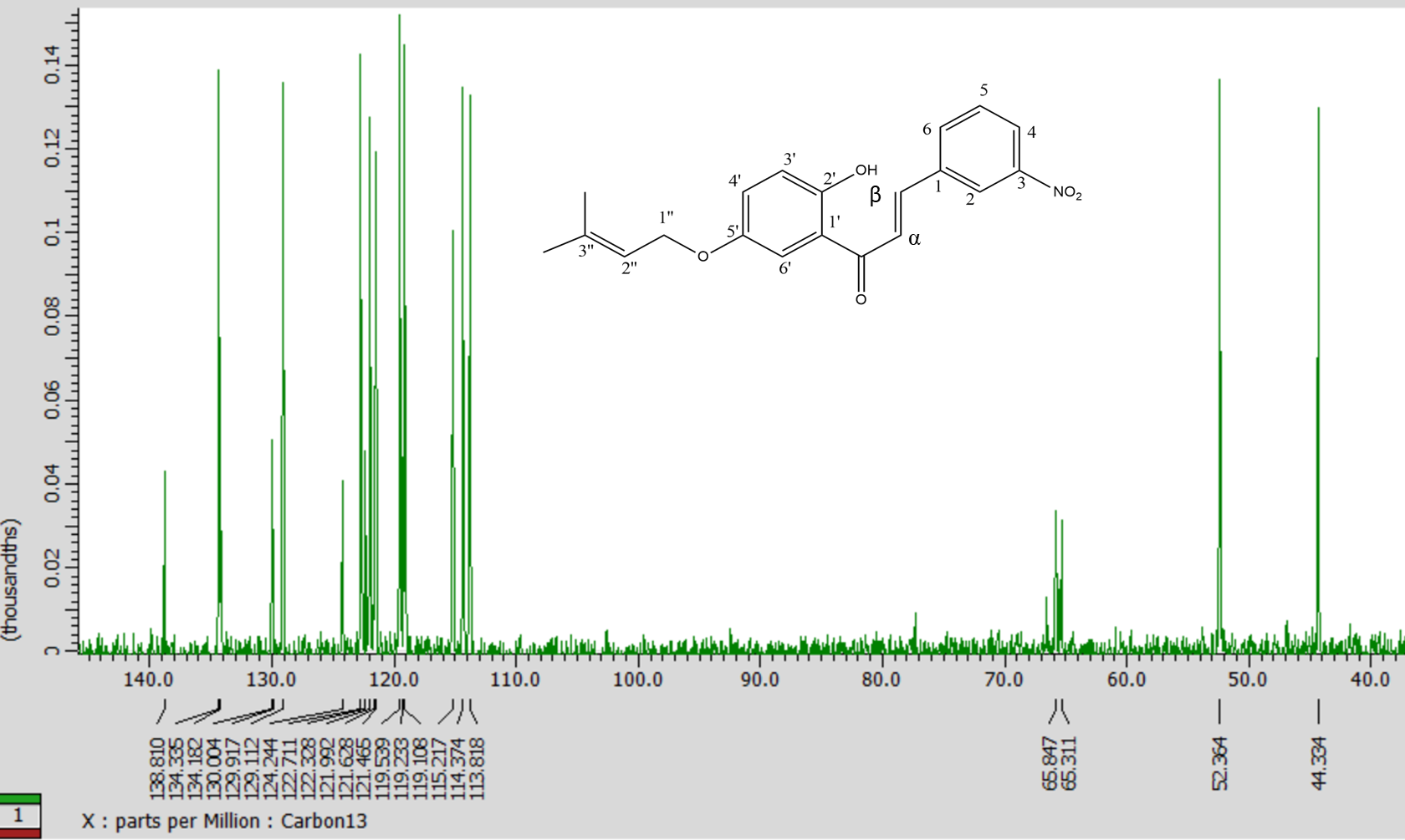


**Figure 7**: DEPT 90 spectrum of compound 5A

**
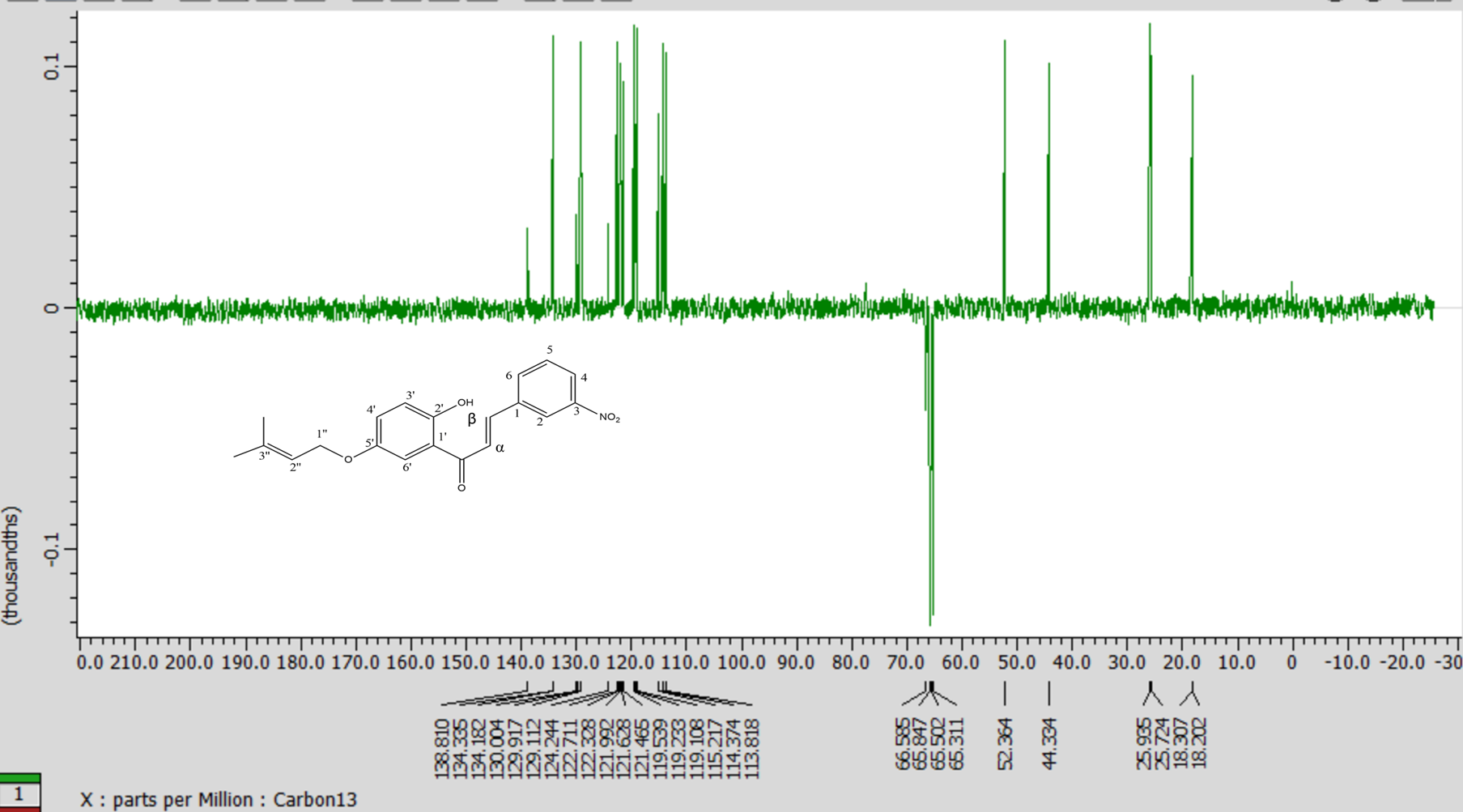
**

**Figure 8**: DEPT 135 spectrum of compound 5A
